# Spin Coherence in a Phosphine Oxide-Based Mononuclear Yb^III^ Complex

**DOI:** 10.1021/acs.inorgchem.6c02812

**Published:** 2026-09-08

**Authors:** Joan Torrent, Oriol Balsells, Radovan Herchel, Cristina Puigjaner, Valentin Novikov, Albert Escuer, Arnald Grabulosa, Júlia Mayans

**Affiliations:** † Departament de Química Inorgànica i Orgànica, Secció de Química Inorgànica, 216515 Universitat de Barcelona, Marti i Franques 1-11, Barcelona 08028, Spain; ‡ Department of Inorganic Chemistry, Faculty of Science, Palacký University, Olomouc 77147, Czech Republic; § Departament de Mineralogia, Cristal·lografia i Dipòsits Minerals and Unitat de Difracció de R-X. Centre Científic i Tecnològic de la Universitat de Barcelona (CCiTUB), Universitat de Barcelona, Solé i Sabarís 1-3, Barcelona 08028, Spain; ∥ Institut de Nanociència i Nanotecnologia (IN2UB), Universitat de Barcelona, Barcelona 08028, Spain

## Abstract

The implementation of lanthanide-based molecular systems in quantum technologies (QTs) requires an accurate and well-designed environment of the spin-bearing lanthanide ion. Lanthanides have become appealing candidates for QTs given their ability to display both slow magnetic relaxation and quantum coherence, in tandem with other properties, which can be achieved through a rational design of the ligand field. This work covers the characterization of a Yb^III^ compound based on phosphine oxide ligand bis­(diphenylphosphino)­methane oxide (dppmO_2_). The compound with a structural formula of [Yb­(dppmO_2_)­(NO_3_)_3_(H_2_O)]·CH_2_Cl_2_ (**1**) was structurally characterized through X-ray diffraction techniques. Field-induced slow magnetic relaxation was detected through AC SQUID magnetometry. The spin Hamiltonian parameters were evaluated through both continuous wave (CW) and echo-detected field swept (EDFS) electron paramagnetic resonance (EPR). The field and temperature dependence of the phase memory time (*T*_m_) and spin–lattice relaxation (*T*_1_) were determined through Hahn echo decays and inversion recovery pulse sequences, respectively. Strong modulations in the Hahn echo decays were rationalized through coupling to ^14^N nuclei present in the nitrato donors, while Rabi oscillations show the ability of the system to behave as an effective qubit. Theoretical calculations employed at the DFT and CASSCF/NEVPT2 level were performed to elucidate the electronic structure and magnetic properties of **1**.

## Graphical abstract

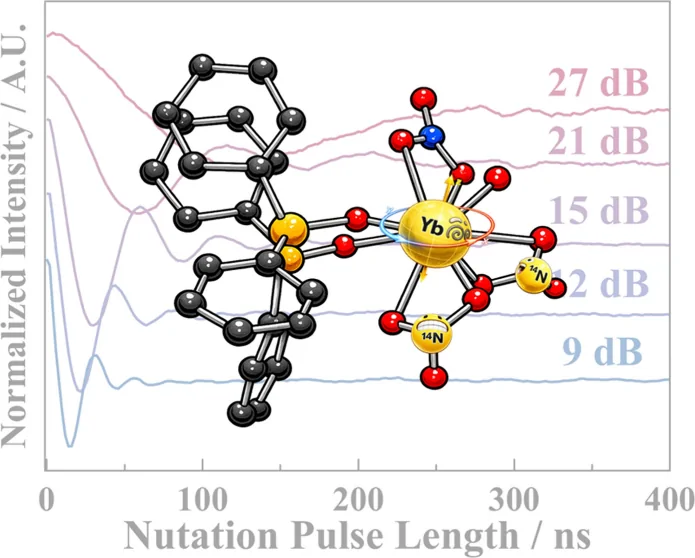

## Introduction

With the rise of quantum computing, molecular spin-based materials have gained considerable attention given the possibility to manipulate their spin manifold through resonant microwave pulses,^1,2^ which are able to place opposite spin orientations into arbitrary and coherent superpositions. Molecular materials have some specific advantages over current state-of-the-art qubits, most of which are based on solid-state materials synthesized through top-down approaches. These top-down synthetic methodologies introduce inevitable atomic-level defects, promoting differences in the quantum coherences of each qubit, which are tremendously sensitive to the environment and to the qubit–qubit interaction. Moreover, the random positions of the qubits in the system forbid individual scalability. Through a bottom-up synthetic route granted by coordination chemistry, molecular systems present a scalable, versatile, and defect-free promising platform, with quantum coherence having been observed up to room temperature^3,4^ and in time scales comparable to other spin-based devices,^5^ such as Nitrogen-Vacancy (NV) in diamond. Furthermore, the intimate correlation between the ligand field and the spin Hamiltonian makes key parameters such as the spin coherence lifetime (denoted through the phase memory time *T*_*m*_), the nuclear spin bath, or the qubit–qubit distance tunable through synthetic chemistry. However, there are still many drawbacks in comparison to other physical realizations such as superconducting systems or polarized photons, mainly related to decoherence rates, which, although being long enough to enable the implementation of quantum gates, are yet shorter than the aforementioned systems.

The first and main molecular approach has been to use systems based on *S* = 1/2 transition metal ions such as Cu^II^,^6–10^ VO^II^,^11–15^ or Ag^II^.^16^ These systems, which possess only one unpaired electron, provide the necessary two-leveled system from which to gain insights into the mechanisms governing quantum coherence and its decay. However, lanthanide-based systems are also attractive candidates, insofar as lanthanide-based coordination complexes have also been proposed for other appealing applications such as luminescence,^17–19^ or magnetic coolants.^20–22^

The most frequently employed lanthanide ion in quantum computing has been trivalent gadolinium,^23–28^ which has shown the longest coherence times. Furthermore, the experimental addressability through conventional X-band Electron Paramagnetic Resonance (EPR) of its full *S* = 7/2 manifold has been proposed as a route to achieve qubits with multiple addressable levels,^29–31^ known as qu*d*its. Nevertheless, other lanthanides have also displayed quantum coherence in molecular systems, such as Er^III^,^20,32,33^ Nd^III^,^28^ Ce^III^,^34^ and Yb^III^.^35^ These lanthanides are characterized by a very strong intrinsic single-ion anisotropy, which stems from the strong Spin–Orbit Coupling (SOC), consequence of the unquenched orbital angular momentum (OAM) that their shielded *4f* electrons yield. As a consequence, under a suitable ligand field, the energy gap between the ground and first excited states can be large enough so as to consider the system as an isolated spin doublet in the same fashion as the thoroughly studied transition metal discussed above, that is to say, the *S*_*eff*_ = 1/2 required to construct the simplest qubit.

This work is devoted to the investigation of the magnetic and spectroscopic properties of a molecular mononuclear Yb^III^ compound. To the best of our knowledge, quantum coherence has only been reported in one Yb^III^-based system, having been thoroughly studied in the prototypical Yb­(trensal) complex and its derivatives.^29,35–40^

For the present work, we utilized a phosphine oxide-based ligand (Figure 1a,b). These ligands have been found to be suitable to achieve lanthanide-based systems displaying slow magnetic relaxation (SRM).^41^ This property is highly desirable in the quest for lanthanide-based qubits, since the presence of SRM denotes a spin–lattice relaxation time (*T*_*1*_) long enough so as not to hinder *T*_*m*_, since *T*_*1*_ sets its upper limit such that *T*_*m*_ ≤ 2*T*_1_. As a result, and given the affinity of these ligands to coordinate to lanthanide ions, the suitability of phosphine oxides to bring about quantum coherence must be evaluated. Furthermore, the lower number of protons in comparison to the benchmark Yb­(trensal) qubit provides a suitable nuclear spin bath in order to enhance quantum coherence. The synthesis of the ligand was performed through the oxidation of the commercial phosphine precursor bis­(diphenylphosphino)­methane (dppm) with hydrogen peroxide, leading to the diphosphine oxide bis­(diphenylphosphino)­methane oxide (dppmO_2_). By reacting one equivalent of Yb­(NO_3_)_3_ · 5H_2_O with one equivalent of dppmO_2_ under aerobic conditions, the mononuclear compound [Yb­(NO_3_)_3_(dppmO_2_)­(H_2_O)]·CH_2_Cl_2_ (**1**) was obtained. Both static and dynamic magnetic susceptibility measurements were performed, the latter revealing a field-induced slow relaxation of the magnetization. Continuous-Wave Electron Paramagnetic Resonance (CW-EPR) was carried out to delve into the electronic structure of **1**, while Pulse EPR measurements were performed to interrogate the spin dynamics of **1**, and reveal quantum coherence, indicated by the presence of Rabi oscillations. Theoretical calculations revealed a large rhombic magnetic anisotropy of the ground state Kramers doublet, which is found to be very well-isolated from excited states in terms of energy gap, and suggest that it can be influenced by the intermolecular interactions.

**Figure 1:**
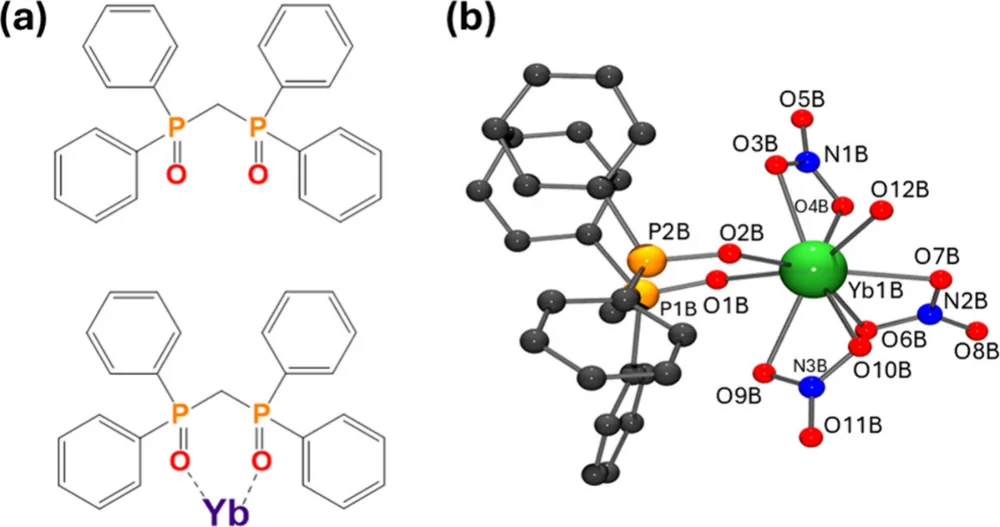
(a) Scheme of the dppmO_2_ ligand employed in this work. (b) Partially labeled plot of the molecular structure of complex **1**.

## Experimental Section

### Materials, Methods, and Physical Measurements

All reagents and solvents were used as received with no further purification. Infrared spectra were recorded in the 4000–400 cm^–1^ range on a Thermo Nicolet scientific IS5 spectrophotometer, with KBr as a dispersing agent and the OMNIC software package for visualization of the spectra. Magnetic susceptibility and magnetization measurements were performed on pressed polycrystalline samples of **1** in the 2–300 K range with a Quantum Design MPMS-XLSQUID magnetometer at the CCiT Magnetochemistry Unit of the University of Barcelona. Diamagnetic corrections were estimated from Pascal tables. CW and Pulse EPR measurements were performed using a Bruker Elexsys E580 spectrometer operating in X-band, equipped with a Stinger cryogen-free variable temperature control system, a Flexline cryostat, and an ER 4118X-MD5 dielectric resonator. All samples were prepared in tubes with a 4 mm outer diameter.

Hahn echo decays were recorded using the sequence: (*π/2*)–*d*_*1*_–(π)–*d*_*1*_–echo, with *d*_*1*_ incremented in 2 ns steps starting from 100 ns. Pulse lengths were 8 ns for π/2 and 16 ns for π. The total envelope of the spin echo was integrated. Echo-Detected Field-Swept (EDFS) spectra were recorded through applying Hahn echo sequences under static field variation. *T*_*m*_ was measured at selected field positions by monitoring the Hahn echo decay signal as a function of *d*_*1*_. The Hahn echo decays were analyzed using nonlinear least-squares fitting. For the temperature-dependent data set, the magnetic field was fixed at 350 mT. For the field-dependent data set, the Hahn echo decays were recorded at 7 K over a range of magnetic fields between 200 and 600 mT.

Rabi oscillations were recorded by applying a nutation microwave pulse of variable length, adding a constant delay of 10 ms much longer than *T*_*m*_ time and monitoring the echo intensity as a function of nutation pulse duration. The nutation pulse width was incremented in steps of 2 ns. Baseline correction (first-order polynomial), exponential windowing function, zero-padding, and Fourier transformation were applied to the time-domain signal. The resulting spectra were analyzed to extract Rabi frequencies. The dependence of the experimental Rabi frequency on the calculated nutation frequency (assuming 44 MHz at 0 dB attenuation) was evaluated to verify the expected microwave power scaling behavior. Spectral simulations were performed using the EasySpin.^42^

### X-ray Crystallography

White prism-like specimens of complex **1** were mounted on a Bruker D8 Venture system equipped with a multilayer monochromator and a Mo microfocus (λ = 0.71083 Å). The frames were integrated with the Bruker SAINT software package using a narrow-frame algorithm. The structure was solved and refined using the Bruker SHELXTL Software Package.^43^ Collection and refinement data are provided in Table S1. PXRD spectra (Figure S1) were performed on a PANalytical X’Pert PRO MPD θ/θ powder diffractometer of 240 mm radius, in a convergent beam configuration with a focalizing mirror and a transmission geometry with flat samples sandwiched between low absorbing films. Cu Kα radiation (λ = 1.5418 Å) was employed with a work power of 45 kV to 40 mA. The scans were performed in the 2–60° 2θ range with a step size of 0.0263° and a measuring time of 300s per step and refined using the Bruker SHELXTL software.

### Computational Details

The quantum-chemical calculations were performed with the ORCA 6.0 computational package.^44,45^ To treat relativistic effects, the Douglas-Kroll-Hess (DKH) Hamiltonian was applied,^46,47^ together with SARC2-DKH-QZVP for lanthanide atoms, DKH-def2-TZVP for all other atoms except for C, H atoms for which DKH-def2-SVP basis was used.^48,49^ Additionally, SARC/J Coulomb fitting basis set^50^ and RIJCOSX approximation were utilized.^51–53^ The largest Integration grid (DefGrid3) was used in all calculations. The molecular structures were extracted from X-ray data with the atomic positions of hydrogen atoms normalized in Mercury software.^54^ Next, the state average complete active self-consistent field (SA-CASSCF)^55^ wave function method was employed, with the active space defined by 13 electrons in seven f-orbitals, CAS­(13e,7o), which resulted in seven doublets. Additionally, the N-electron valence second-order perturbation (NEVPT2) was applied to CASSCF wave functions.^56^ The SINGLE_ANISO module was applied to calculate the magnetization reversal barrier and magnetic data.^57,58^ The DFT calculations were accomplished with ωB97x-V range-separated hybrid functional^59^ complemented by a self-consistent nonlocal dispersion correction (SCNL) and using the same basis sets as for CASSCF calculations. The calculated electron densities were analyzed with AIMAII software.^60^

### Synthetic Procedures

#### Bis­(diphenylphosphino)­methane Dioxide (dppmO_2_)

To a stirred solution of bis­(diphenylphosphino)­methane (5.2 mmol, 2 g) in 15 mL of THF, a 30% H_2_O_2_ solution was added dropwise (9.8 mmol, 1 mL). Due to the highly exothermic nature of this process, the reaction was carried out in an ice bath. The solution was allowed to stir for 30 min. The solution was filtered to obtain the ligand as a white powder in quantitative yields. IR (KBr, cm^–1^): 3400 (s), 3060 (m), 2950 (mw), 1640 (mw), 1500 (vs), 1400 (ms), 1380 (m), 1290 (vs), 1160 (vs), 1030 (m), 791 (mw), 690 (mw), 569 (w), 507 (mw). ^1^H NMR (CDCl_3_, δ ppm) = 7.69–7.63 (m, 8H); 7.37–7.19 (m, 12H), 3.46 (t, J_HP_ = 16 Hz, CH_2_). ^31^P­{^1^H} NMR (CDCl_3_, δ ppm): 24.65 (s), which nicely agrees with the published data.^61^

#### Synthesis of **1**

To a stirred solution of dppmO_2_ (0.4 mmol, 0.170 g) in acetonitrile (20 mL), Yb­(NO_3_)_3_ · 5H_2_O (0.180 g) was added. The reaction mixture was stirred overnight and evaporated to dryness to obtain a white crystalline powder, which was recrystallized from dichloromethane. Single crystals suitable for X-ray diffraction were obtained through the slow evaporation of a CH_2_Cl_2_ solution of **1**. IR spectrum is shown in Figure S2. CNH for C_25_H_24_N_3_O_12_P_2_Yb, calc/found: C, 37.84/37.77; H, 3.05/3.09; N, 5.30/5.24.

## Results and Discussion

### Structural Description

Compound **1** crystallizes in the *P*2_1_/*c* space group (Table S1). The unit cell contains two crystallographically nonequivalent molecules (labeled as atoms A or B), which show similar but not identical environment. The crystal packing is shown in Figure S3–S5 and selected bond distances and angles are shown in Table S2. A labeled plot of **1A** is shown in Figure 1b. The enneacoordinated sphere of the Yb^III^ cation is fulfilled with one bidentate dppmO_2_ ligand, three chelating nitrato ligands and one water molecule, resulting in an overall YbO_9_ environment. SHAPE^65^ analysis reveals that the closest structural match for the “A” molecule is a monocapped square antiprism (CShM = 2.14) and a muffin polyhedron for the “B” molecule (CShM = 2.05) (Figure S6). However, the coordination environment is severely distorted from the corresponding ideal polyhedron due to the low bite angle of the bidentate nitrato ligands that promote O–Yb–O bond angles close to 53°. The Yb–O_nitrato_ bond distances are longer than the Yb–O-donors from the dppmO_2_ and H_2_O ligands.

The coordinated water molecules establish a set of double H bonds with the nitrato ligands from their neighboring molecules resulting in a supramolecular 2D arrangement of complexes, with YbA···YbB distance of 7.159 Å and YbA···YbA’ and YbB···YbB’ of 6.658 and 6.680 Å respectively. The obtained structure radically differs from the structure obtained from the ligand dppeO_2_ (1,2-bis­(diphenylphosphino)­ethane oxide) counterpart, reported recently in previous works.^64^ The methylene bridge of the dppmO_2_ ligand does not allow for the bridging-like coordination motif obtained with dppeO_2_. As a result, we obtained zero-dimensional systems with significantly different properties.

### Magnetic Properties

#### Static Measurements

Static magnetic susceptibility measurements were taken on pressed polycrystalline samples of **1** (Figure S7). The room temperature *χ*_*M*_T value for **1** is 2.62 cm^3^ mol^–1^ K in the experimental 2.4–3.0 cm^3^ mol^–1^ K usual range expected for an isolated Yb^III^ cation (^2^F_**7**/2_). Due to the depopulation of the Stark sublevels, that occurs upon lowering temperature, the *χ*_*M*_T plot decreases continuously down to 1.35 cm^3^ mol^–1^ K at 2 K. The magnetization measurement reaches the 1.72 *N*_β_ quasi saturated value under the maximum explored field of 5 T, in good agreement with the usual values found in the literature. Susceptibility and magnetization data were analyzed by means of CASSCF/NEVPT2 calculations (see below).

#### Dynamic Measurements

Preliminary Alternate Current (AC) susceptometry measurements under a 3G oscillating field at a fixed frequency of 1000 Hz reveal no out-of-phase magnetic susceptibility signal at zero-field, which is related to a fast and effective Quantum Tunnelling of the Magnetization (QTM) in between the ground Kramers’ doublet (KD) (Figure S8). This fact is supported by the relatively high tunnelling transition probability obtained in the ab initio calculations (see below). Upon applying a static dc field as low as 0.02 T, the degeneracy between the ground state KD is lifted, suppressing QTM and enabling an out-of-phase signal to arise. As the field is increased, the intensity of the signal increases, but the position of the maxima remains constant. Between 0.5 and 0.7 T the signal remains the same, after which, at higher fields the intensity starts to decrease, but there is yet no temperature shift on the maxima, indicating a nondependence of the relaxation rate (τ) on the static magnetic field (Figure S8) in the studied field range. Further measurements were performed at a static field of 0.5 T (Figure 2a) at variable temperature in the 10–1488 Hz frequency range. At low frequencies, only weak tails can be appreciated which have fully decayed at 4 K. As the frequency is increased, maxima start to appear, which slightly shift to higher temperatures as the frequency is further increased. The highest temperature maximum corresponds to the 1488 Hz frequency, and is located at about 4 K. At 8 K, there is no more out-of-phase component at any frequency. Out-of-phase maxima are also present in the frequency-dependent plots (Figure 2b), which are located close to the highest frequencies measured. From the Cole–Cole plot (Figure 2c), the temperature dependence of the relaxation rate (τ) (Figure 2d) can be extracted by fitting the data to the generalized Debye model.^62–64^

1 $$\tau^{-1}={\tau_{QTM}}^{-1}+CT^{n}$$

where the first and second terms correspond to QTM and Raman relaxation pathways, respectively. The presence of two different relaxation pathways operating in distinct temperature regimes is further reinforced by the two slopes observed in the log–log plot (Figure S9). The obtained parameters from the fit are summarized in Table S3. The fitting values are in good agreement with those expected and present in the literature.

The presence of a signal in the out-of-phase regime implies that the spin–lattice relaxation time (*T*_1_) is long enough so as not to hinder spin–spin relaxation (*T*_2_), which is in essence the time during which our system retains quantum coherence, making **1** a promising candidate as a molecular spin qubit.

**Figure 2:**
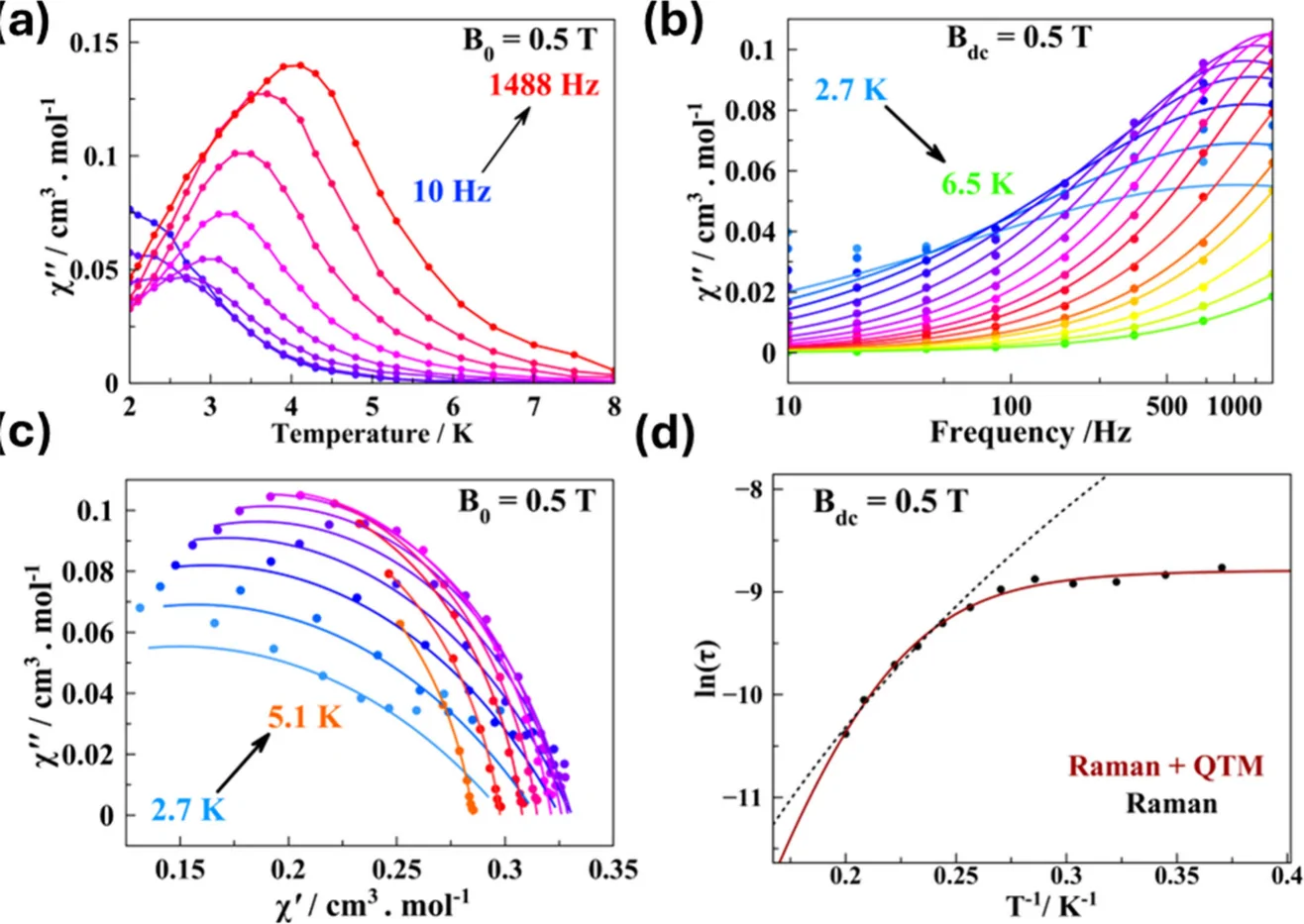
(a) Temperature and (b) frequency dependencies of the out-of-phase magnetic susceptibility component of **1**. (c) Argand plot of **1**. (d) Temperature dependence of the relaxation rate of **1**. The lines correspond to the best fits, except for those of panel a, which are guides for the eye.

#### Electron Paramagnetic Resonance

To delve into the electronic structure of **1**, X band CW-EPR was taken on a polycrystalline sample of **1** at 4 K, diluted with its isostructural Y^III^ analogue at a doping level of 10% (**1**_**10%**_) (Figure 3, top) The CW-EPR spectrum shows two main signals, a single peak at 125 mT and a wide 5-fold split feature between 300 and 400 mT. The spectrum was fit employing the Hamiltonian in eq 2:

2 $$Η=\mu_{B}SgB+ÎAŜ$$

where the first and second term in eq 2 correspond to the Zeeman and hyperfine interactions, respectively. ***B*** is the applied magnetic field, *μ*_*B*_ is the Bohr magneton, *g* is the effective Landé *g*-tensor, and *I* and *S* are the nuclear and electronic spins, respectively, the latter herein considered as effective *S* = 1/2 in the spectral simulations. The two *g*-values were determined to be *g*_*y*_ = 1.98 and *g*_*z*_ = 5.48, which are in good agreement with those obtained from theoretical calculations (Table S4). According to such calculations, the value of *g*_*x*_ is below 0.46 in any monomer or supramolecular dimer, and hence it is out of the available magnetic field range, since a magnetic field above 1500 mT would be required to observe resonance (X band). There is a clear splitting of the spectral line in *g*_*y*_ which is due to hyperfine interactions to the two nuclear spin-active Yb isotopes: ^171^Yb and ^173^Yb. This hyperfine coupling is also observed in *g*_*z*_ as a line asymmetry. Spectral simulations reveal hyperfine coupling tensors of [*A*_*y*_, *A*_*z*_] = [299.99, 299.58] and [261.51, 313.28] for ^171^Yb and ^173^Yb, respectively. We could not determine any hyperfine interaction along *g*_*x*_ due to its absorption being out of the commercial X-band experimental magnetic field range.

**Figure 3:**
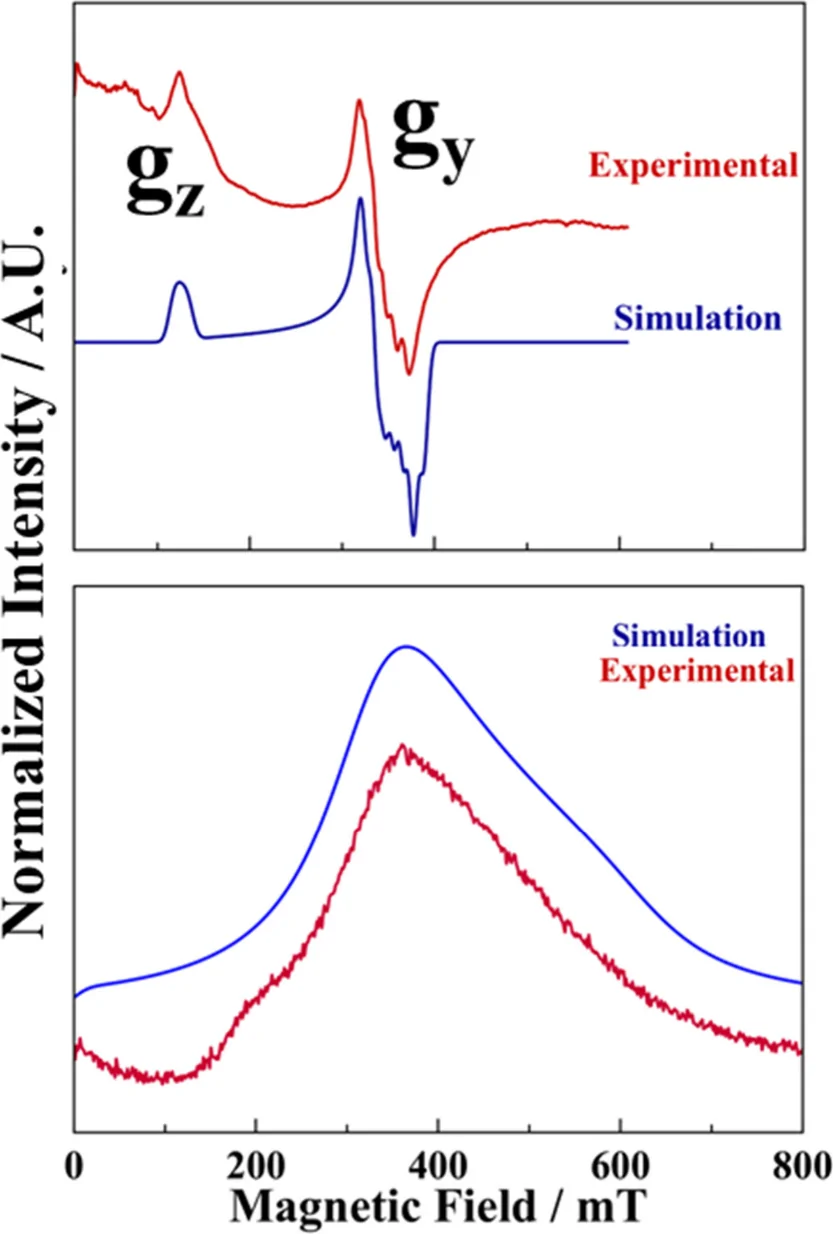
Experimental and simulated CW (top) and EDFS (bottom) EPR spectra of **1**_**10%**_ recorded at X-band frequency (9.8 GHz).

To further delve into the quantum coherent properties of this system, pulse-EPR was performed at X-band in the diluted **1**_**1%**_ sample in the 0–800 mT field range at 7 K (Figure 3). The Echo-Detected Field Swept (EDFS) spectrum of **1** shows a broad absorption spanning from 150 to 1000 mT. The absorption peak appears at 350 mT, which is consistent with *g*_*y*_ in the CW spectrum. Furthermore, an absorption elbow is found at 150 mT, and is ascribed to *g*_*z*_. Noteworthily, the features found in the EDFS spectrum are heavily dependent on the interpulse delay time (Figure S10), which is indicative of significant Echo Envelope Modulation (ESEEM) effects. These effects, which are not present in the CW regime, are usually justified as the reason behind small shifts in CW and EDFS spectra. Furthermore, we observe that the first derivative of the EDFS spectrum (Figure S10) shows *g*_*y*_ at the same resonance position as the CW-EPR, while *g*_*z*_ is shifted upfield in the EDFS spectrum, component which slightly shifts to higher fields and whose intensity vanishes upon increasing the interpulse delay time. This behavior is indicative of faster spin–spin relaxation in the *g*_*z*_ direction than along *g*_*y*_.

Both field and temperature dependence of the spin–lattice relaxation times (*T*_*1*_) were monitored through inversion recovery experiments in the 200–600 mT and 4–11 K field and temperature ranges, respectively (Figure S11, S13). *T*_*1*_ was determined through fitting the inversion recovery time traces to the stretched monoexponential:

3 $$I=I_{0}+e^{-{\left(\frac{\tau_{p}}{T_{1}}\right)}^{\beta_{1}}}$$

The *T*_*1*_ values obtained at different fields and temperatures are summarized in Tables S5 and S6. The temperature dependence of *T*_*1*_ was reliably fit only through the Raman term in eq 1 (Figure 4, top). At these dilution ratios and magnetic fields, spin–spin interactions are too quenched to induce QTM. At the lowest measured temperature (5 K), the *T*_*1*_ value is 4-fold larger than that obtained through ac SQUID magnetometry. Such a discrepancy is a further indication of the dipolar-interaction driven magnetic relaxation taking place in **1**_**100%**_ through QTM. The *n* value obtained from the fitting is of 8.53(2), which is in very good agreement with the one obtained via SQUID magnetometry (8.8(4), Table S3). The field dependence of *T*_*1*_ (Figure 4, bottom) shows an increase in *T*_*1*_ up to about 350 mT, which is the maximum position in the EDFS spectrum, as the field is moved out of resonance, the noise in the inversion recovery signal starts increasing and the *T*_*1*_ value starts to decrease.

**Figure 4:**
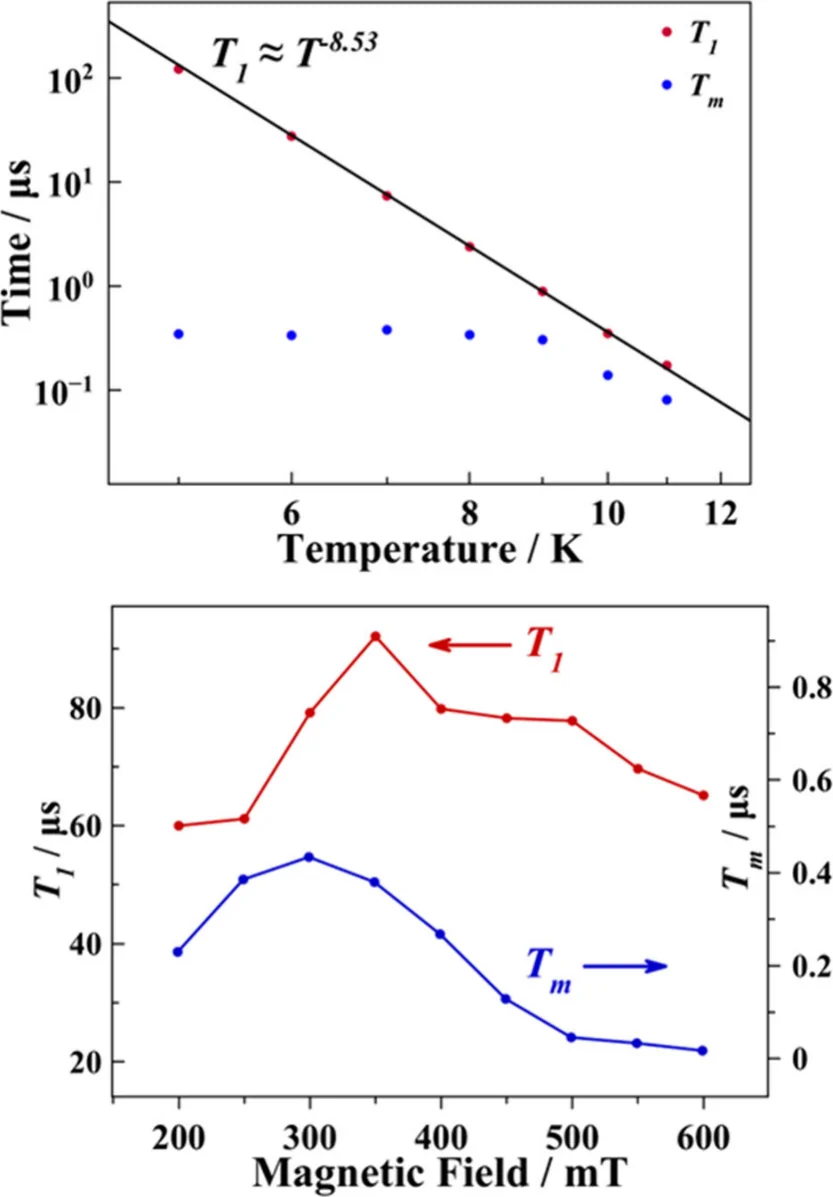
Temperature (top) and field (bottom) dependencies of the spin–lattice relaxation time (*T*_*1*_) and the phase memory time (*T*_*m*_).

To interrogate and quantify the quantum coherent properties of this system, we monitored the Hahn echo decay of **1**_**1%**_ as a function of the magnetic field and temperature (Figures S12, S14) in the 200–600 mT and 5–11 K ranges. The time traces show a very strong modulation, which is a consequence of ESEEM effects mediated by nearby nuclei through hyperfine coupling. As a result, fitting the decay traces merely through stretched exponential functions yields to unreliable phase memory time (*T*_*m*_) values. Consequently, two cosine modulation functions were added, resulting in fitting to eq 4:

4 $$I=I_{0}+Ae^{-{\left(\frac{2\tau }{T_{m}}\right)}^{\beta_{m}}}\left[1+m_{1}\;\cos\left(2\pi f_{1}\tau +\varphi_{1}\right)+m_{2}\;\cos\left(2\pi f_{2}\tau +\varphi_{2}\right)\right]$$

where the first term is the stretched exponential term, and the second term accounts for the observed modulations, each with a specific amplitude (*m*), phase (φ), and frequency (*f*_*i*_). The obtained values for the fitting procedures at each temperature and field position are summarized in Tables S7 and S8. To avoid overparametrization, the initial *A*, *β*_*m*_, and *T*_*m*_ values were set from the nonmodulated fitting, and the modulation frequencies were estimated from Fourier transforms. Furthermore, the two obtained frequencies from fitting across field positions were fixed in the fitting of the temperature dependent traces. We found that *T*_*m*_ remains constant across low temperatures (Figure 4, top), with a value of about 0.40 *μs*, which favorably compares to the 0.22 *μs* coherence time determined for the Yb­(trensal) system at these temperature range. This difference, even at the higher dilution ratio recorded for **1**_**1%**_, might be due to the lower proton number in the dppmO_2_ ligand in comparison to trensal.^39^

Such a constant dependence at these temperature where spin–lattice relaxation is not limiting *T*_*m*_ is indicative that quantum coherence is being quenched by the interaction between the Yb electron spin and nearby nuclei, such as ligand protons or ^14^N nitrogen nuclei (see below). As *T*_*1*_ plummets at temperatures above 9 K due to the *T* ^*n*^ dependence of the Raman relaxation channel, *T*_*m*_ starts to decrease accordingly, given that *T*_*1*_ sets the upper limit for *T*_*m*_ such that *T*_*m*_ ≤ 2*T*_*1*_. Furthermore, the modulation is still present as the temperature is increased, and is clearly visible until the highest measured temperature of 11 K.

The field dependence of *T*_*m*_ (Figure 4, bottom) shows a similar trend to that of *T*_*1*_, showing an increase up to the most intense position in the EDFS spectrum and decreasing when the field is moved out of resonance, with a maximum *T*_*m*_ value of 0.43 *μs* obtained at 300 mT. Apart from *T*_*m*_, other parameters which show a strong field dependence are the two modulation frequencies: From their field evolution, the nuclei driving these modulations along with their hyperfine coupling can be inferred following:

5 $$f\left(B\right)=\sqrt{{\left(\gamma B+s\right)}^{2}+q^{2}}$$

where γ was fixed to the gyromagnetic ratio of the ^14^N nuclei (3.044 MHz/T), and *s* is the longitudinal component of the hyperfine coupling, further modulated by the transverse *q* component. The two frequencies were reliably fit to eq 5 (Figure 5) to obtain hyperfine coupling values of [*A*_∥_*N*1__, *A*_⊥_*N*1__] = [-0.79(1) −0.17(2)] and [*A*_∥_*N*2__, *A*_⊥_*N*2__] = [0.17(2) 0.25(6)].

**Figure 5:**
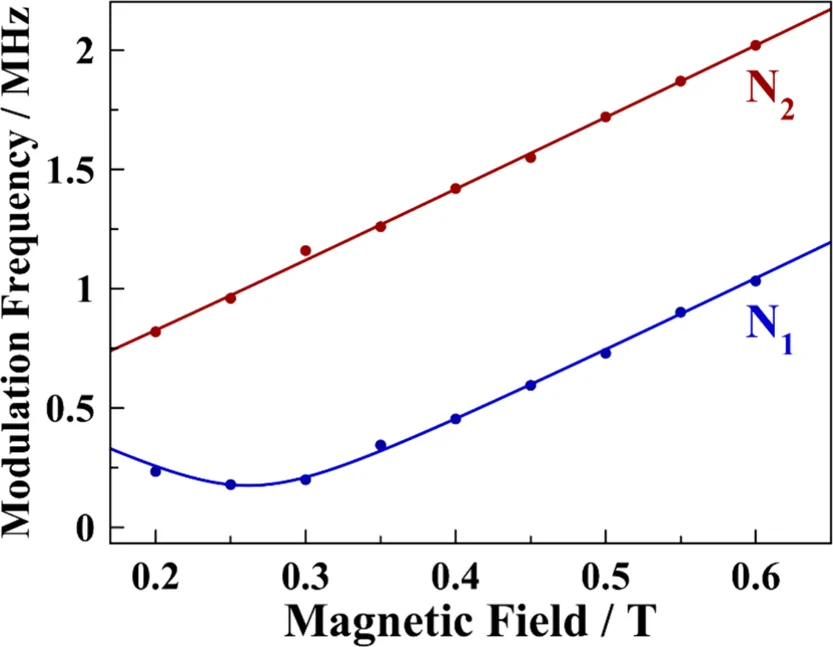
Field dependence of the two modulation frequencies obtained by fitting the Hahn echo decays to cosine modulated stretched exponential functions. The solid lines correspond to the best fits to eq 5.

To determine whether the observed quantum coherence allows for a coherent and arbitrary spin manipulation of **1**, we performed transient nutation experiments at X-band. We observed Rabi oscillations in a wide range of microwave power attenuation values (Figure 6a). The observation of these Rabi oscillations, and the expected linear relationship between the Rabi frequency and the microwave nutation frequency (Figure 6b,c) prove that the spin system can be placed in any arbitrary linear combination between its two opposite spin projections.

**Figure 6:**
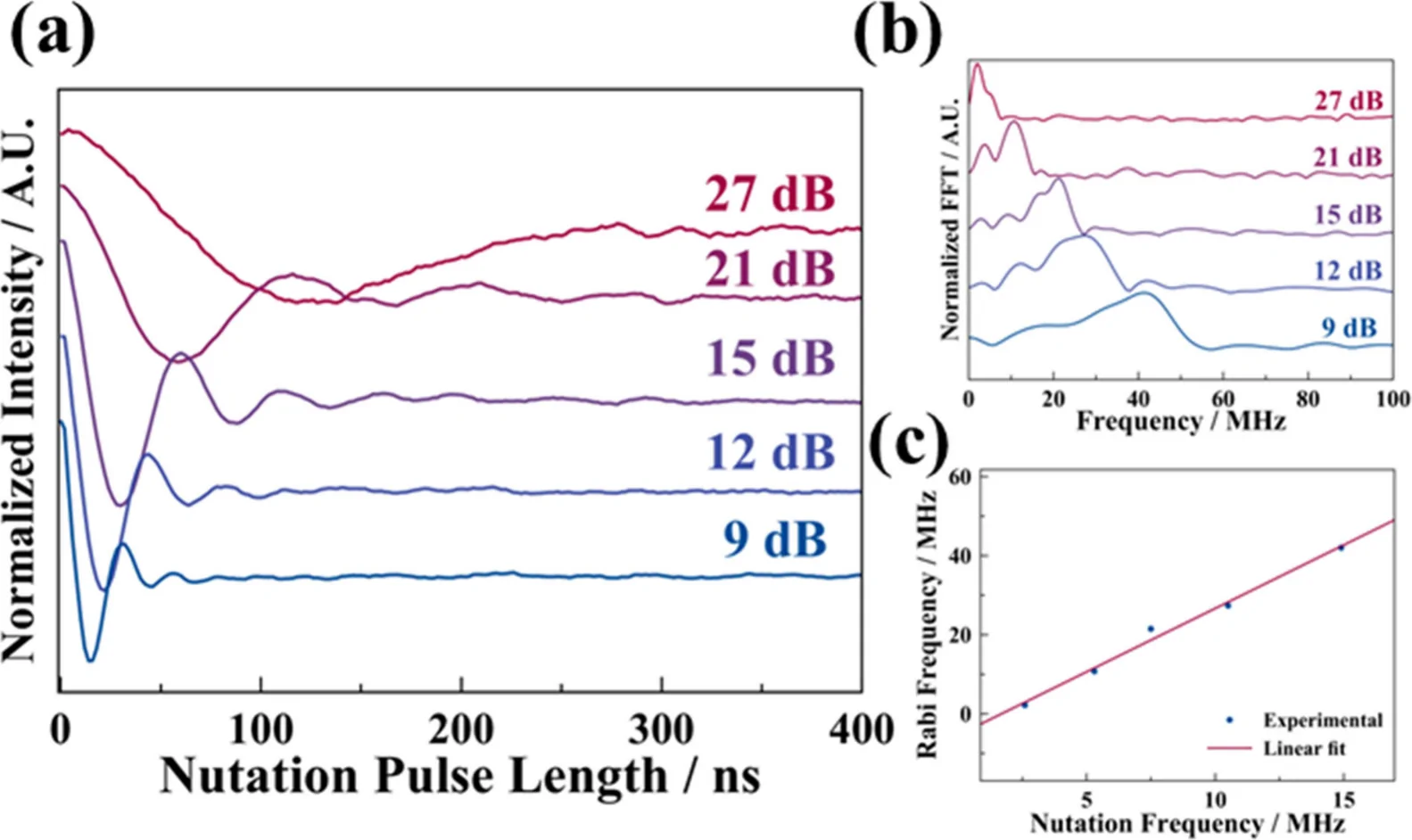
(a) Rabi oscillations of **1** recorded under a static field of 350 mT at 5 K at different microwave power attenuations. (b) FFT of the Rabi oscillations of **1**. (c) Rabi frequency dependence on the nutation frequency. The solid line corresponds to the best linear fit.

### Computational Results

As there are two crystallographically independent Yb^III^ complexes, A and B, we initially conducted the CASSCF/NEVPT2 calculations for both. However, the inspection of the crystal structure revealed the formation of a 1D supramolecular chain of these complexes, and therefore, we decided to explore the impact of the second-coordination sphere and the intermolecular interactions on the electronic and magnetic properties of **1**. Hence, the CASSCF/NEVPT2 calculations were also performed for supramolecular dimers of the type AA, BB, and AB. Such calculations were done by replacing one paramagnetic Yb^III^ ion with a diamagnetic Lu^III^ ion, resulting in labeling A­(AA), B­(BB), A­(AB), B (AB), where the first letter corresponds to the Yb atom in the given supramolecular dimer written in parentheses. The methodology employed in these calculations is elucidated in the Experimental Section.

The respective energies of f-orbitals and the ligand field multiplets originating from ^2^F_7/2_ for Yb^III^ ions are shown in Figure 7. The calculated energy of the first excited Kramers doublet (KD) is 123 cm^–1^ for A and 149 cm^–1^ for B of **1**. The results for the supramolecular dimers show larger variation, from the value of 123 cm^–1^ for A­(AA) up to the value of 209 cm^–1^ for B­(AB). The magnetization reversal barriers calculated by the SINGLE_ANISO module are depicted in Figure 7b for monomers and in Figure S15 for supramolecular dimers. Intermolecular interactions also influence the calculated probability of the quantum tunnelling of the ground state, and it is evident that these probabilities are lower in supramolecular dimers (0.0206 – 0.132) than in monomers (0.223 – 0.255). Hence, it seems that the formation of the 1D supramolecular chain in 1 can lead to slowing down the relaxation of the magnetization, at least by the quantum tunnelling process. In spite of that, the calculated probabilities for the quantum tunnelling of the magnetization of the ground state are still sufficiently high, thereby necessitating a modest static field to observe a gradual relaxation of the magnetization, a finding that aligns with the AC susceptibility experiments. The orientation of the *g*-tensor axis of the ground state KD is illustrated in Figure S16 for A and B. The easy axis is approximately aligned through two nitrato-ligands bonded in the opposite sites to the Yb central atom. The key parameters of ground-state KD for both monomers and supramolecular dimers are summarized in Table S4. More detailed information about each Kramers doublet can be found in Table S11 (*g*-tensors, decomposition of the wave function into |*J,M*_*J*_ > contributions, and mutual orientation of *g*_*z*_-vectors of KDs). It is interesting to note that in the case of A­(AA) and A­(AB) calculations, the first excited KD changes the type of magnetic anisotropy from axial to planar type, as can be deduced from their *g*-factors. In all other cases, the calculations provided all KDs with axial magnetic anisotropy. It demonstrates that intermolecular interactions affect the first and second coordination spheres^66–69^ and thus can also alter the slow relaxation properties of SMMs. Furthermore, the comparison to the experimental DC magnetic data is illustrated in Figure S7, in which good agreement is found with field-dependent magnetization data, whereas temperature-dependent data show larger discrepancies.

**Figure 7:**
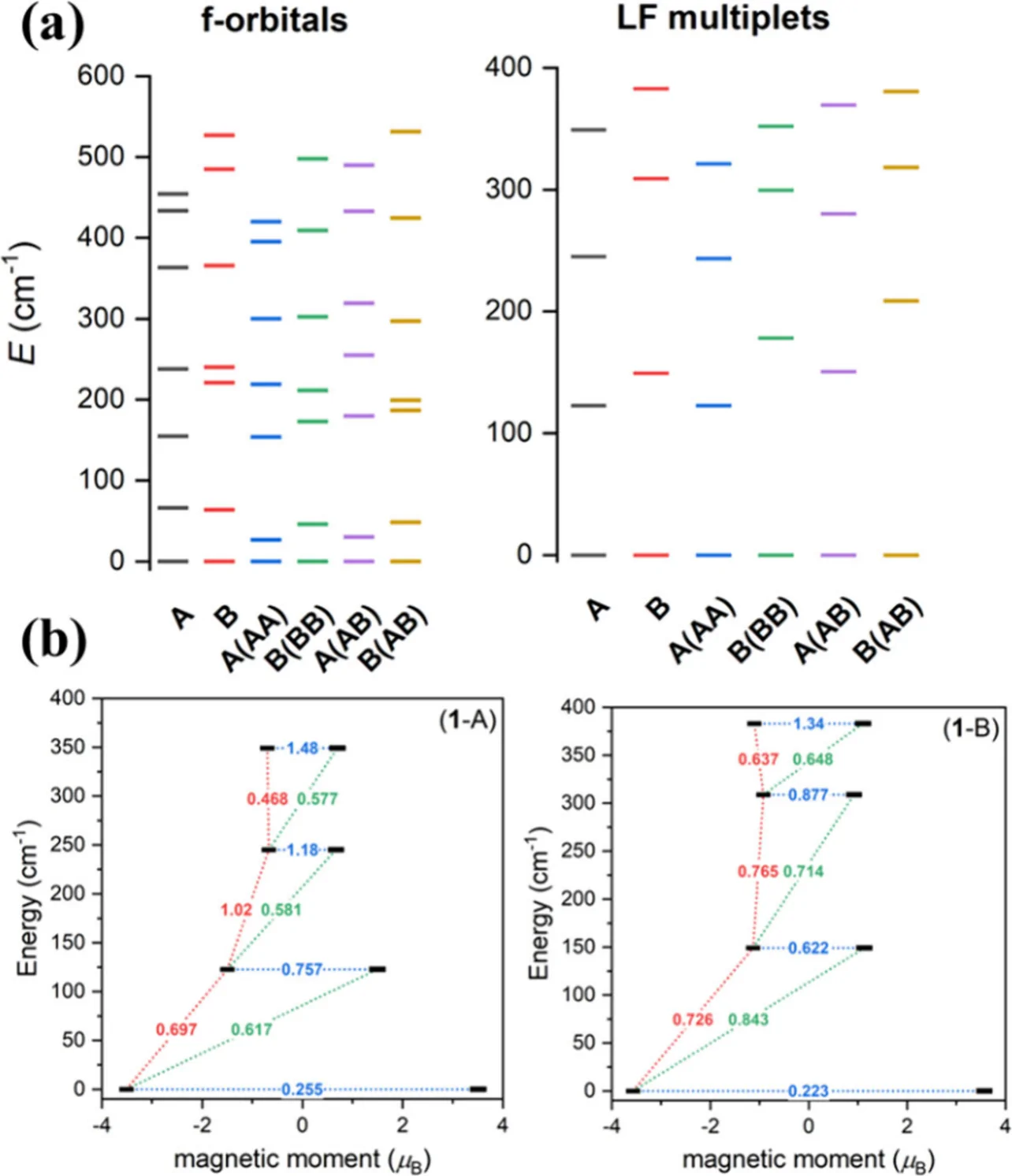
Output of the CASSCF/NEVPT2 calculations for the mononuclear and dinuclear molecular fragments of Yb^III^ compound **1**. (a) AILFT f-orbitals (left) and ligand-field multiplets (LFM) (right) stemming from the ^2^F_7/2_ state for Yb^III^ ions. (b) Magnetization reversal barriers.

Furthermore, our recent work on Ce^III^ and Nd^III^ nitrato-complexes^70^ revealed that in the former complex, semicoordination was found. This inspired us to perform analogous QTAIM analysis (QTAIM = quantum theory of atoms in molecules)^71,72^ using DFT method to provide the information about electron density ρ­(**r**). The results derived with AIMAll software are reported in Tables S9 and S10 for mononuclear complexes A and B of **1**, respectively. Shortly, the critical points of the type (3,–1), also known as bond critical points (BCPs), were identified, and several characteristic functions (the Laplacian of the electron density (∇^2^ρ­(**r**)), electron kinetic energy (*G*(**r**)), potential energy density (*V*(**r**)) and electron energy density (*h*_e_(**r**)) were calculated at these BCPs. For all Yb–O bonds, it holds that (∇^2^ρ­(**r**)) > 0, *h*_e_(**r**) < 0, and |*V*(**r**)|/*G*(**r**) > 1,^73^ which implies that all oxygen donor atoms of nitrato, aqua, and dppmO_2_-ligands are coordinated. However, the ratio |*V*(**r**)|/*G*(**r**) is very close to 1.000 for longer Yb–O bonds of nitrato-ligands and interestingly also for aqua ligand, suggesting that we are at the edge of the coordination - semicoordination border.

## Conclusions

In this work, we have synthesized a novel Yb^III^-based compound (**1**) from a bis-phosphine dioxide ligand (dppmO_2_). This compound has been structurally characterized through X-ray diffraction, which shows a 9-fold coordination of the spin carrier, which lies under a strongly distorted C_4v_ symmetry. Compound **1** shows a field-induced slow magnetic relaxation, which seems to be field-independent under the recorded fields. The system relaxes through a combination of Raman and Quantum Tunnelling of Magnetisation relaxation pathways. The spin Hamiltonian parameters were evaluated through CW and EDFS-EPR. The EDFS spectra at different interpulse delay times were found to be very different, which is ascribed to the strong ESEEM modulation by nearby nuclei. This modulation can also be seen in the Hahn echo decay experiments, wherein from the field dependence of the two determined modulation frequencies two different hyperfine coupling tensors to ^14^N atoms from the nitrato donor were inferred. The field dependence of *T*_*1*_ and *T*_*m*_ shows a similar trend for both times, with maxima corresponding to the maximum positions in the EDFS spectrum. The temperature dependence of *T*_*1*_ shows the same relaxation behavior as that determined through SQUID magnetometry, albeit without the dipolar-induced QTM pathway. *T*_*m*_ shows a constant behavior with temperature, and starts to decrease once it becomes *T*_*1*_-limited. Rabi oscillations were found upon recording transient nutation pulse sequences. The linear dependence of the Rabi frequency with the nutation frequency shows that the system can effectively behave as a qubit, with the superposition between the two resonant states being arbitrarily controllable. Theoretical calculations show that the reported molecular system possesses a large rhombic magnetic anisotropy of the ground Kramers doublet with a relatively large separation of the first excited state (>120 cm^–1^). Hence, this agrees with the interpretation of AC susceptibility data for which direct and Raman processes dominate at low temperatures. To the best of our knowledge, this work represents the second Yb^III^-based system to display both a slow relaxation of the magnetization and quantum coherence, both desirable properties when searching to engineer molecular systems for Quantum Information Technologies.

## Supplementary Material



## Notes

Additional synthetic, structural, magnetic, and spectroscopic data (PDF)
