# Supplementary material for: Spin Coherence in a Phosphine Oxide-Based Mononuclear YbIII Complex
Source: Inorg Chem. 2026 Sep 8;65(37):21649–58. doi: 10.1021/acs.inorgchem.6c02812 (PMC13599388; doi:10.1021/acs.inorgchem.6c02812)
Supplement: Supplementary file 1 [file ic6c02812_si_001.pdf]

Supporting Information

"Spin Coherence in a Phosphine Oxide-Based Mononuclear Yb^{III} Complex."

Joan Torrent,[a] Oriol Balsells[a], Radovan Herchel[c], Cristina Puigjaner[d], Valentin Novikov[a,b], Albert Escuer[a,b], Arnald Grabulosa[a,b], and Júlia Mayans[a,b]*

a Departament de Química Inorgànica i Orgànica, Secció de Química Inorgànica, Universitat de Barcelona, Marti i Franques 1-11, Barcelona-08028, Spain.

b Institut de Nanociència i Nanotecnologia (IN^2UB), Universitat de Barcelona, Barcelona-08028, Spain.

c Department of Inorganic Chemistry, Faculty of Science, Palacký University, Olomouc 77147, Czech Republic; orcid.org/0000-0001-8262-4666

d Departament de Mineralogia, Cristal·lografia i Diposits` Minerals and Unitat de Difracció de R-X,Centres Científics i Tecnològics de la Universitat de Barcelona (CCiTUB). Barcelona 08028, Spain

Corresponding Author E-mail: julia.mayans@ub.edu

Table of contents

1- Structural Information

Table S1: Crystallographic data for the X-ray structure of **1**.

Figure S1: PXRD spectra.

Figure S2: IR spectrum of compound **1**.

Figures S3-S5: Packing of **1** along the a, b, and c crystallographic axes.

Table S2: Selected bond lengths and angles for compound **1**.

Figure S6: Coordination polyhedra around the crystallographically independent Yb1A and Yb1B moieties.

2- DC data

Figure S7: Magnetization and χ_T *vs* *T* plots. Comparison between experimental data and results from theoretical calculations.

3- AC data

Figure S8: Temperature dependence of the in-phase and out-of-phase magnetic susceptibility components recorded at different fields.

Figure S9: Log-log plot for compound **1**.

Table S3: Best obtained fittings with their corresponding standard deviations of the *ac* data to Eq. 1.

4- Electron Paramagnetic Resonance

Table S4. The results of CASSCF/NEVPT2 calculations for the molecular fragments of Yb.

Figure S10: EDFS spectra of $\mathbf{1}_{1\%}$ recorded at a fixed temperature of 7 K and with different interpulse delay time variations.

Figure S11: Inversion recovery experiments recorded at different fields and at a fixed temperature of 7 K.

Figure S12: Hahn echo decays recorded at variable magnetic fields at a fixed temperature of 7 K.

Figure S13: Inversion recovery curves recorded at different temperatures under a static magnetic field of 350 mT.

Figure S14: Hahn echo decay curves recorded at different temperatures under a static field of 350 mT.

Table S5: Values obtained from fitting inversion recovery curves recorded at different fields at a fixed temperature of 7 K to Eq. 3.

Table S6: Values obtained from fitting inversion recovery curves recorded at different temperatures under a static field of 350 mT to Eq. 3.

Table S7: Values obtained from fitting the Hahn echo decays recorded at different fields and at a fixed temperature of 7 K to Eq. 4.

Table S8: Values obtained along with standard deviations from fitting the Hahn echo decays recorded at different temperatures and at a fixed static field of 350 mT to Eq. 4. The asterisks denote fixed parameters in the fitting.

5- Theoretical Calculations

Figure S15: The output of the CASSCF/NEVPT2 calculations for the dinuclear molecular fragments of Yb(III) compound **1** - the magnetization reversal barriers.

Figure S16: The molecular structures of A (left) and B (right) fragments of **1** overlaid with the principal axes of ground state Kramers' doublet g-tensor (x/y/z-axes colored as red/green/blue arrows) resulting from CASSCF/NEVPT2 calculations.

Table S9: The results of QT-AIM analysis for molecular fragment A of **1**

Table S10: The results of QT-AIM analysis for molecular fragment B of **1**

Table S11: Results of CASSCF/NEVPT2 calculations for molecular geometries of **1**.

1- Structural information

Table S1: Crystallographic data of the X-ray structure of **1.**

	**1**
**Formula**	$C_{25}H_{24}N_3O_{12}P_2Yb$
**FW**	793.46
**System**	Monoclinic
**Space group**	P 1 21/c 1
**a/Å**	22.7372(11)
**b/Å**	18.2721(8)
**c/Å**	16.1508(6)
**α/deg.**	90
**β/deg.**	102.388(2)
**γ/deg.**	90
**V/Å3**	6553.7(5)
**Z**	8
**T, K**	100(2)
**λ(MoKα), Å**	0.71073
**ρ_{calc}, g·cm^{-3}**	1.608
**μ(MoKα), mm^{-1}**	0.959
**F (000)**	1581
**R**	0.0698
**ωR^2**	0.1386

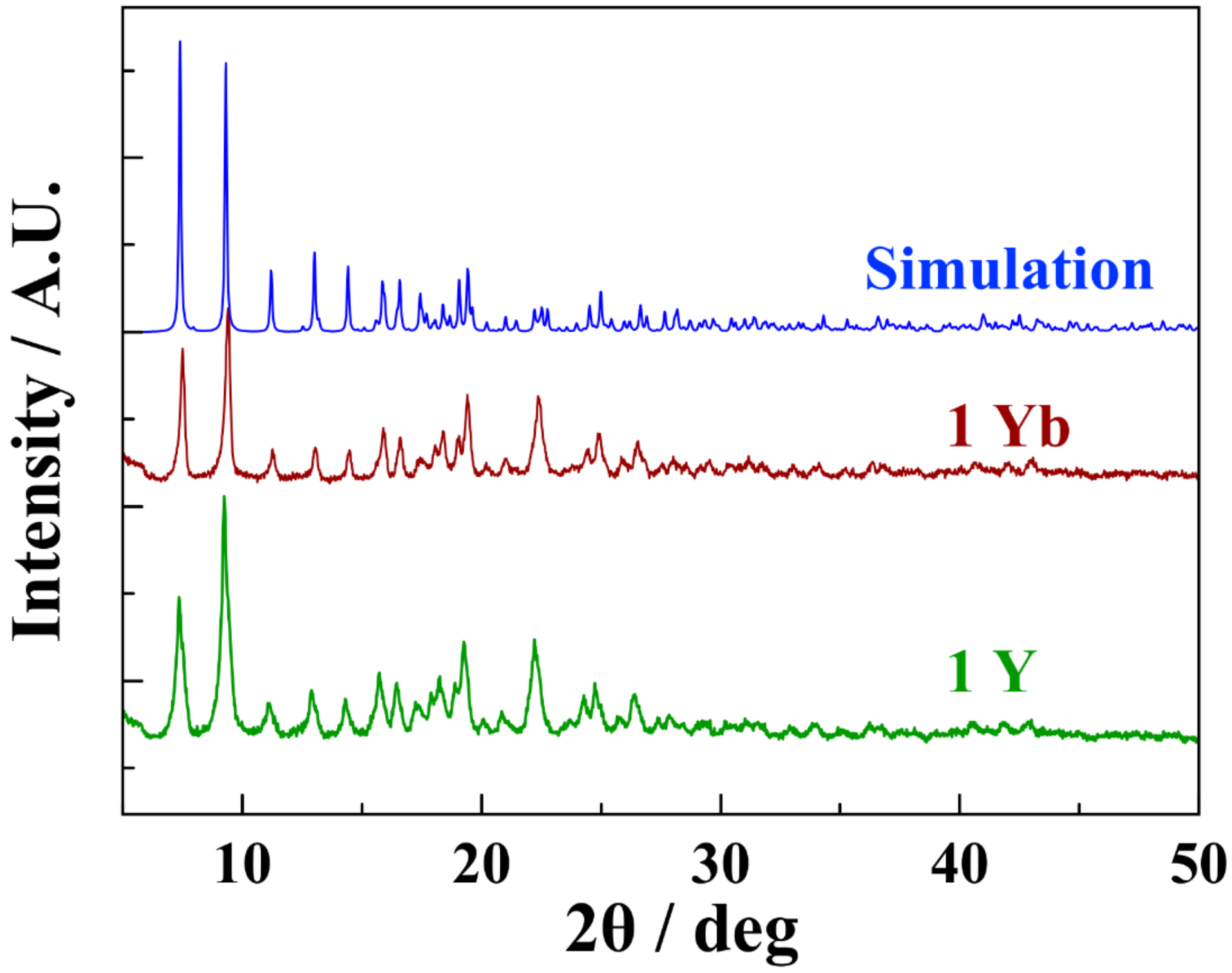

Figure S1: PXRD pattern of compounds **1Yb** and its diamagnetic analogue **1Y**.

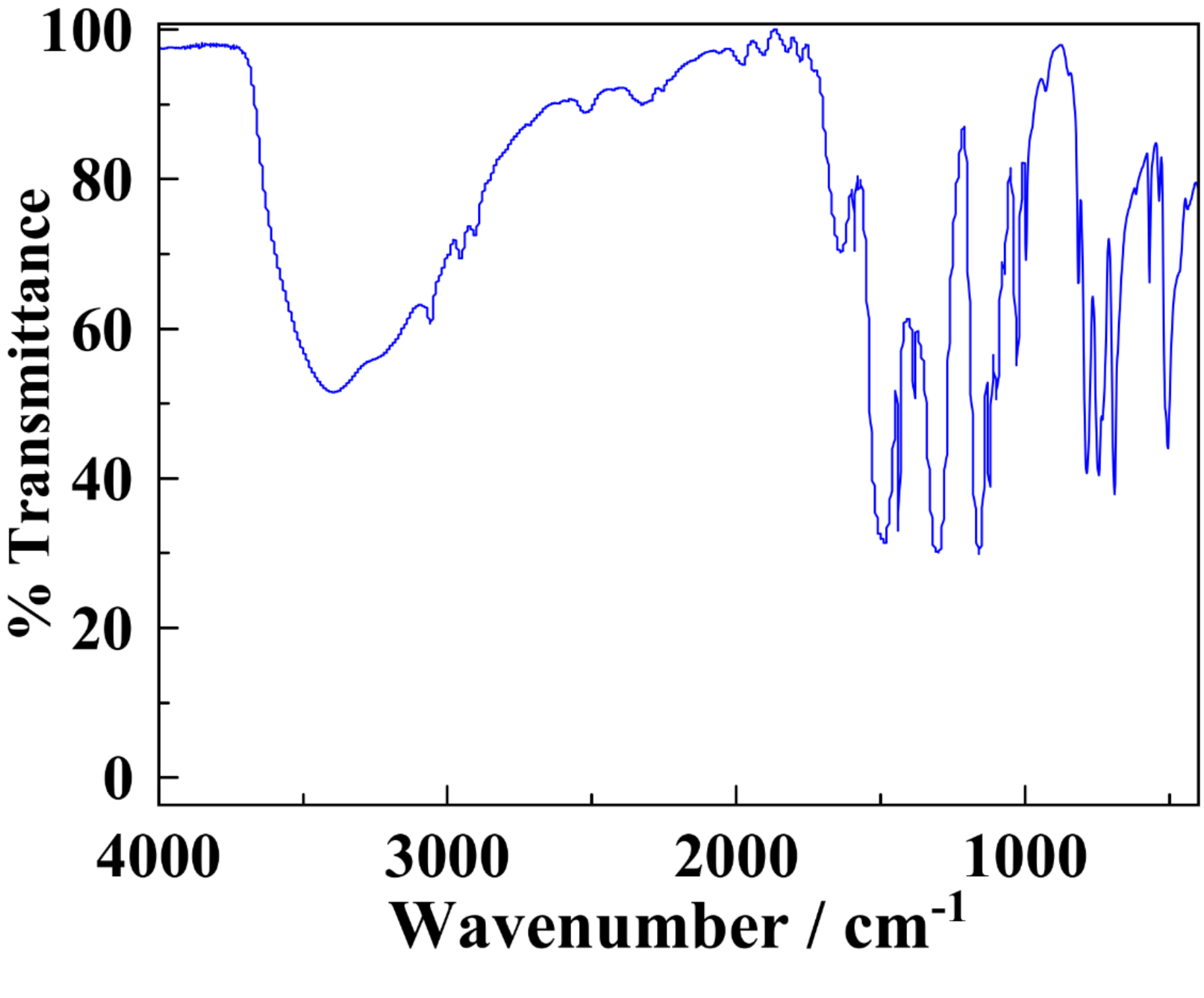

Figure S2: Infrared spectra of compound **1**.

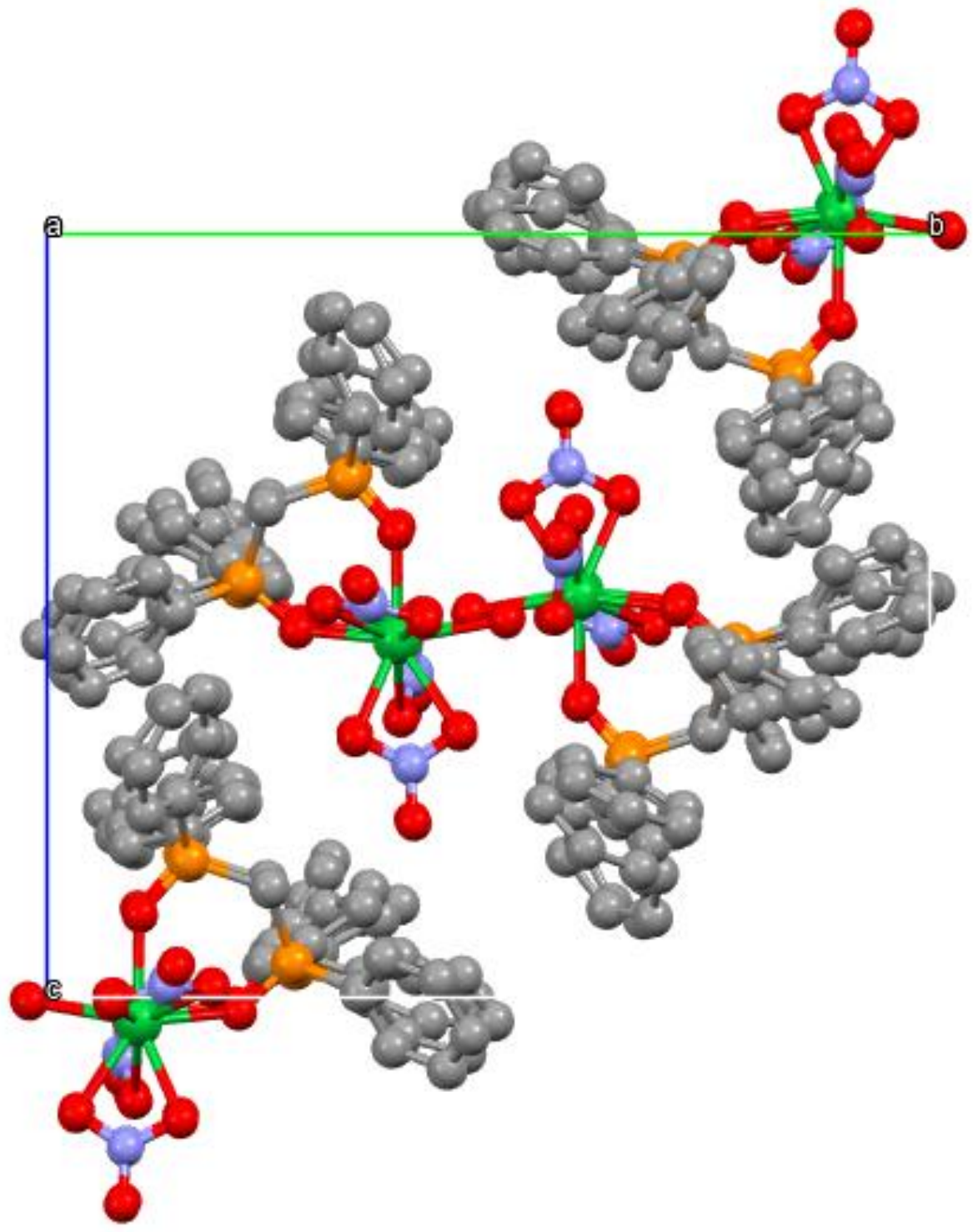

Figure S3: Crystal packing of **1** along the ***a*** crystallographic axis. Color code: Gray: Carbon; Purple: Nitrogen; Red: Oxygen; Orange: Phosphorous; Green: Ytterbium.

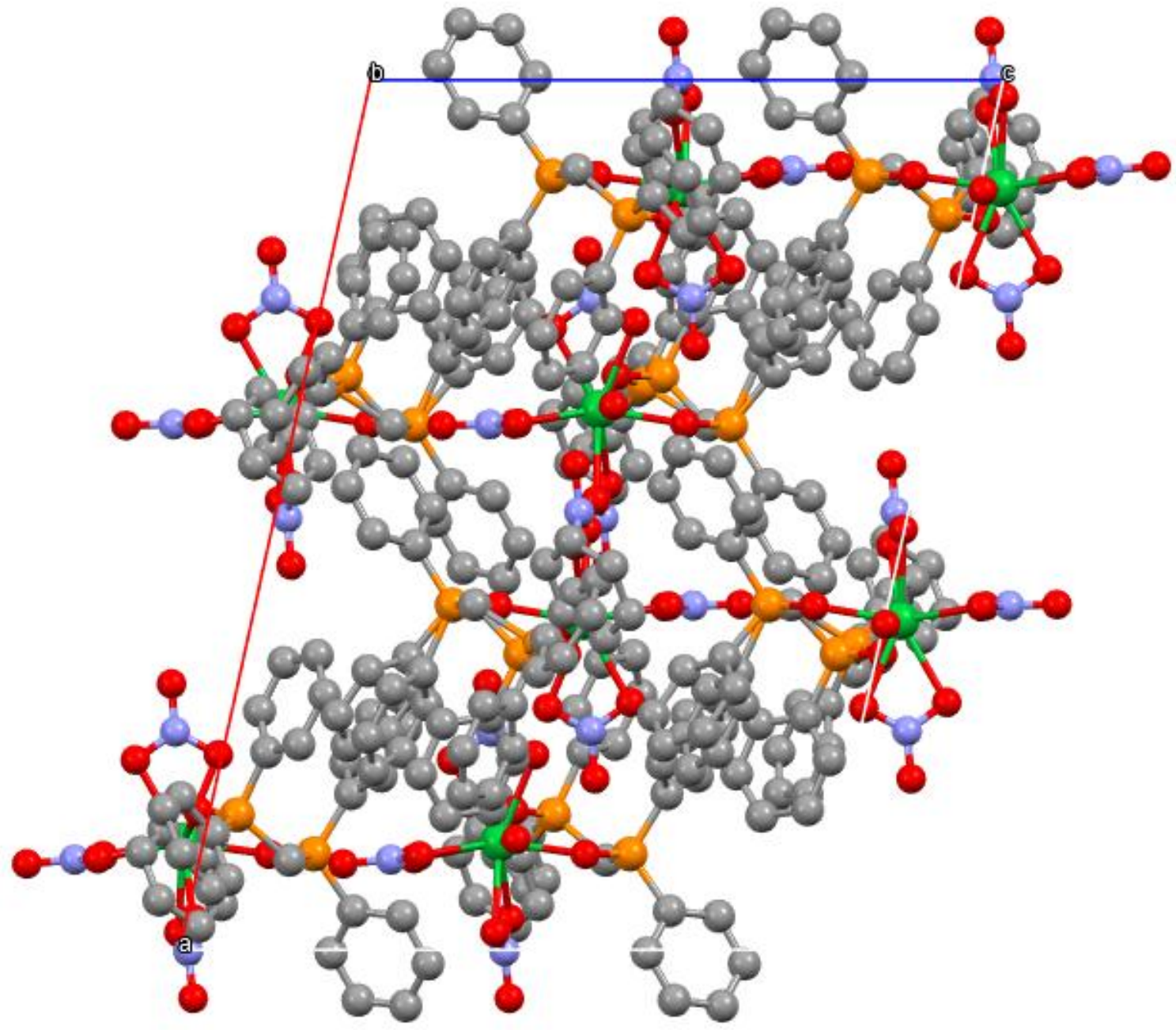

Figure S4: Crytal packing of **1** along the ***b*** crystallographic axis. Color code: Gray: Carbon; Purple: Nitrogen; Red: Oxygen; Orange: Phosphorous; Green: Ytterbium.

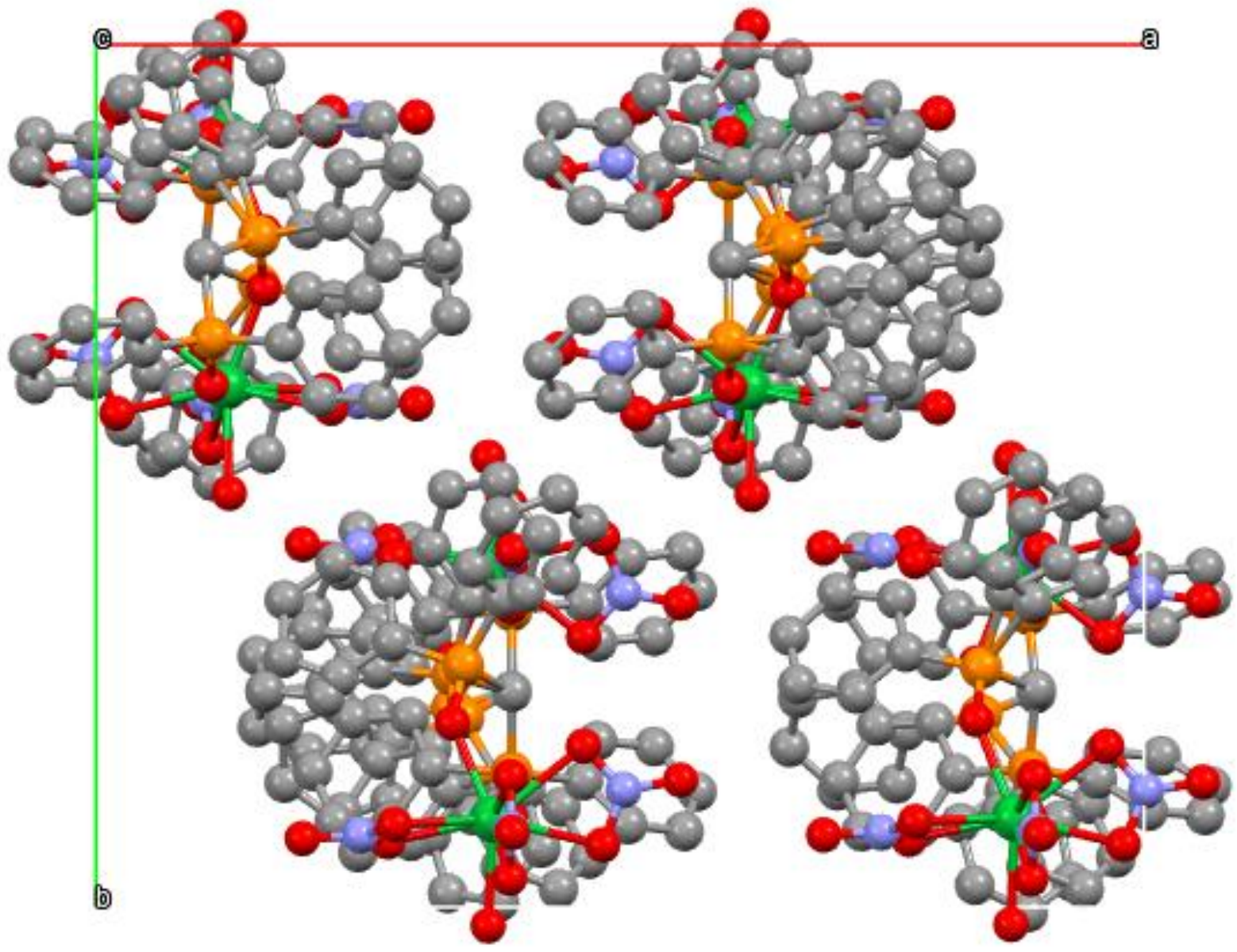

Figure S5: Crytal packing of **1** along the ***c*** crystallographic axis. Color code: Gray: Carbon; Purple: Nitrogen; Red: Oxygen; Orange: Phosphorous; Green: Ytterbium.

Table S2: Selected bond distances (Å) and angles (º) for compound **1**.

Yb1A-O1A	2.230
Yb1A-O2A	2.252
Yb1A-O3A	2.478
Yb1A-O4A	2.392
Yb1A-O6A	2.444
Yb1A-O7A	2.381
Yb1A-O9A	2.463
Yb1A-O10A	2.405
Yb1A-O12A	2.295
O1A-Yb1A-O2A	82.08
O1A-Yb1A-O3A	76.92
O3A-Yb1A-O4A	52.60
O3A-Yb1A-O6A	106.81
O6A-Yb1A-O7A	53.09
O9A-Yb1A-O10A	52.51
O9A-Yb1A-O12A	72.91
O1A-Yb1A-O12A	81.45
O2A-Yb1A-O12A	147.29
Yb1A-O1A-P1A	139.85
Yb1A-O1A-P2A	138.08
P2A-C25A-P1A	113.84

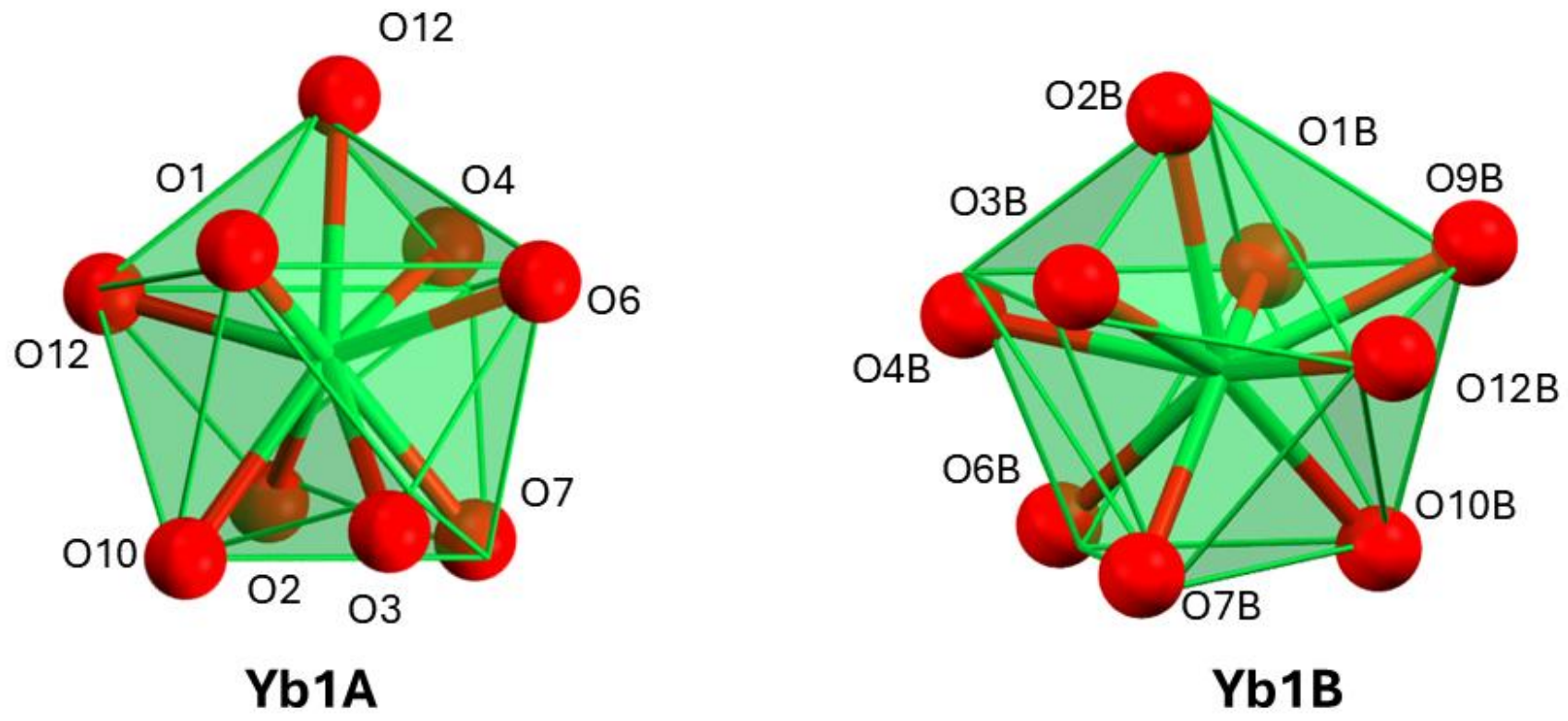

Figure S6: Coordination polyhedra around the crystallographically independent Yb1A and Yb1B moieties.

2- DC data

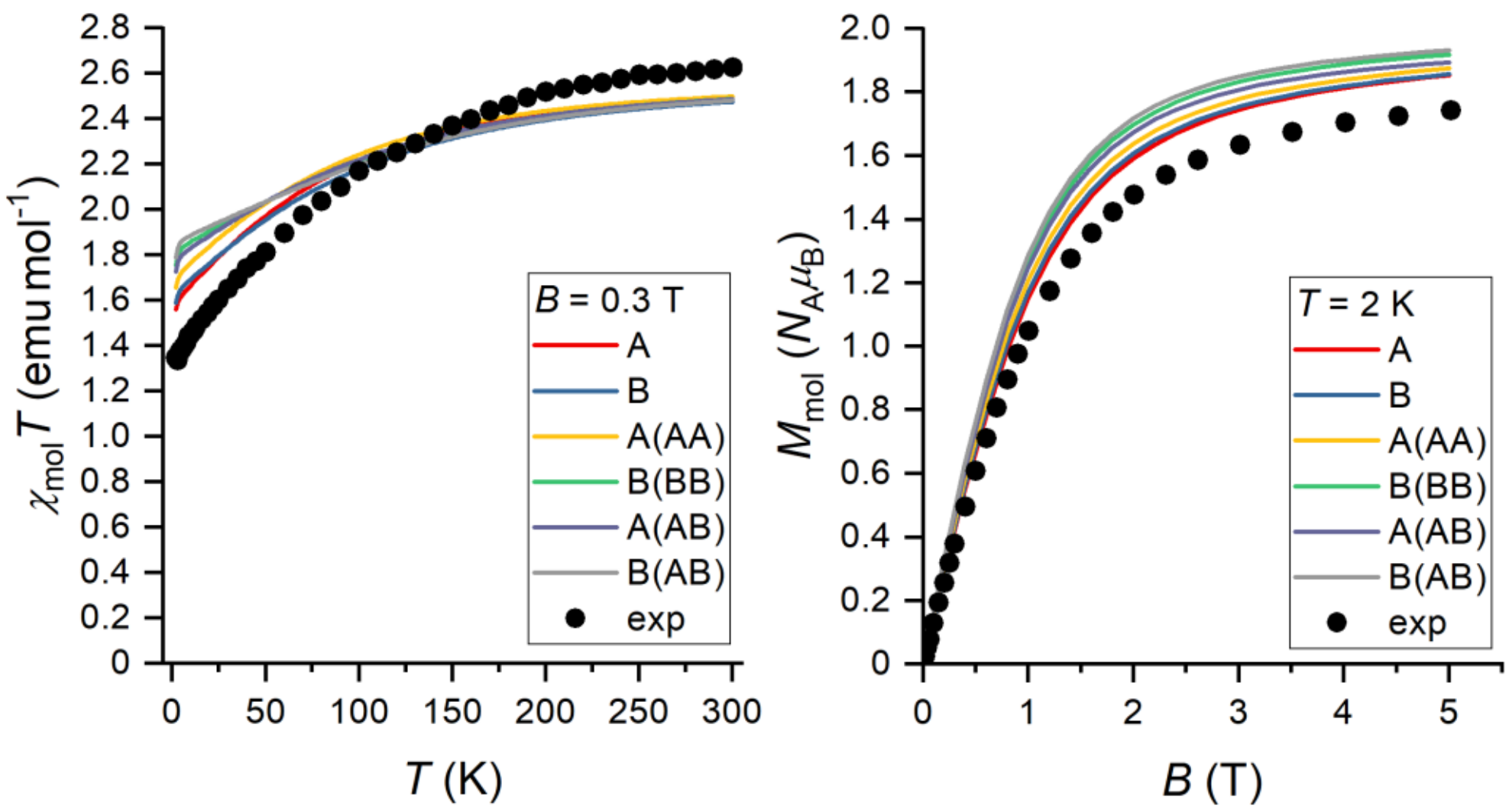

Figure S7: Left, $\chi_m T$ vs T product, comparison between the CASSCF/NEVPT2 calculation outputs and experimental data. Right, M vs B plot, comparison between experimental and CASSCF/NEVPT2 output.

3- AC data

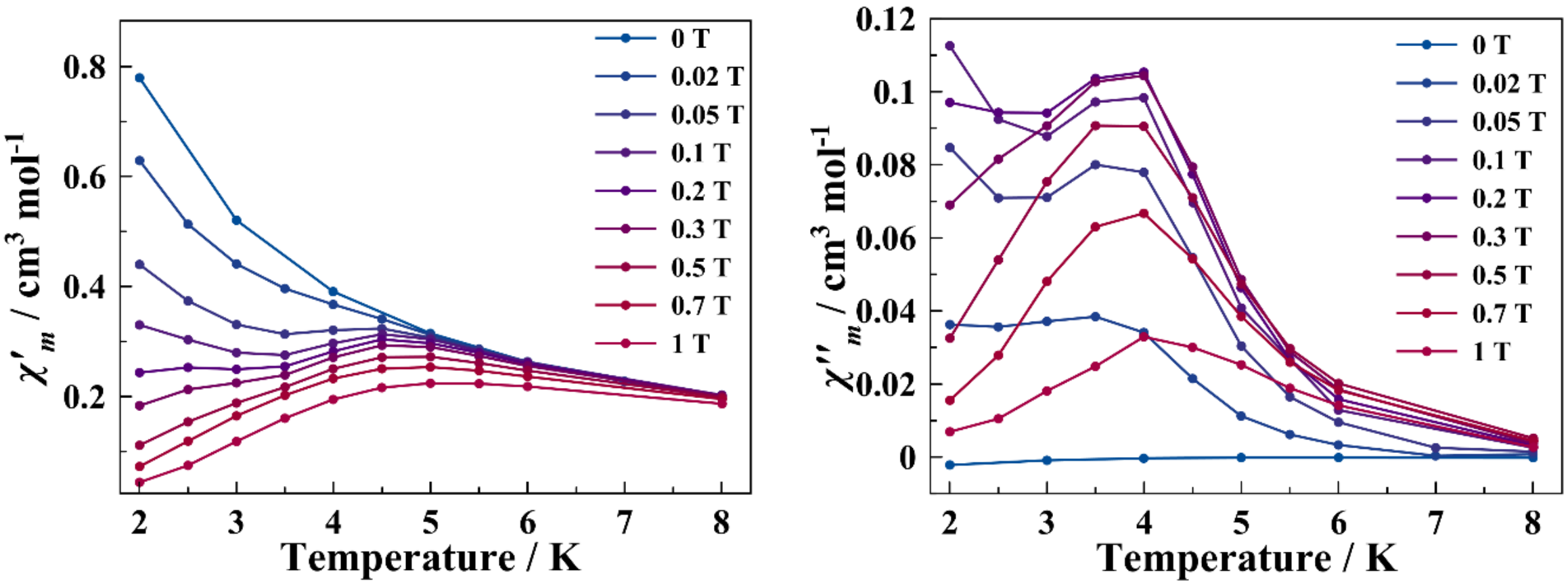

Figure S8: Temperature dependence of the in-phase (left) and out-of-phase (right) components of the magnetic susceptibility in **1**. Recorded at different applied DC fields. The lines are guides for the eye.

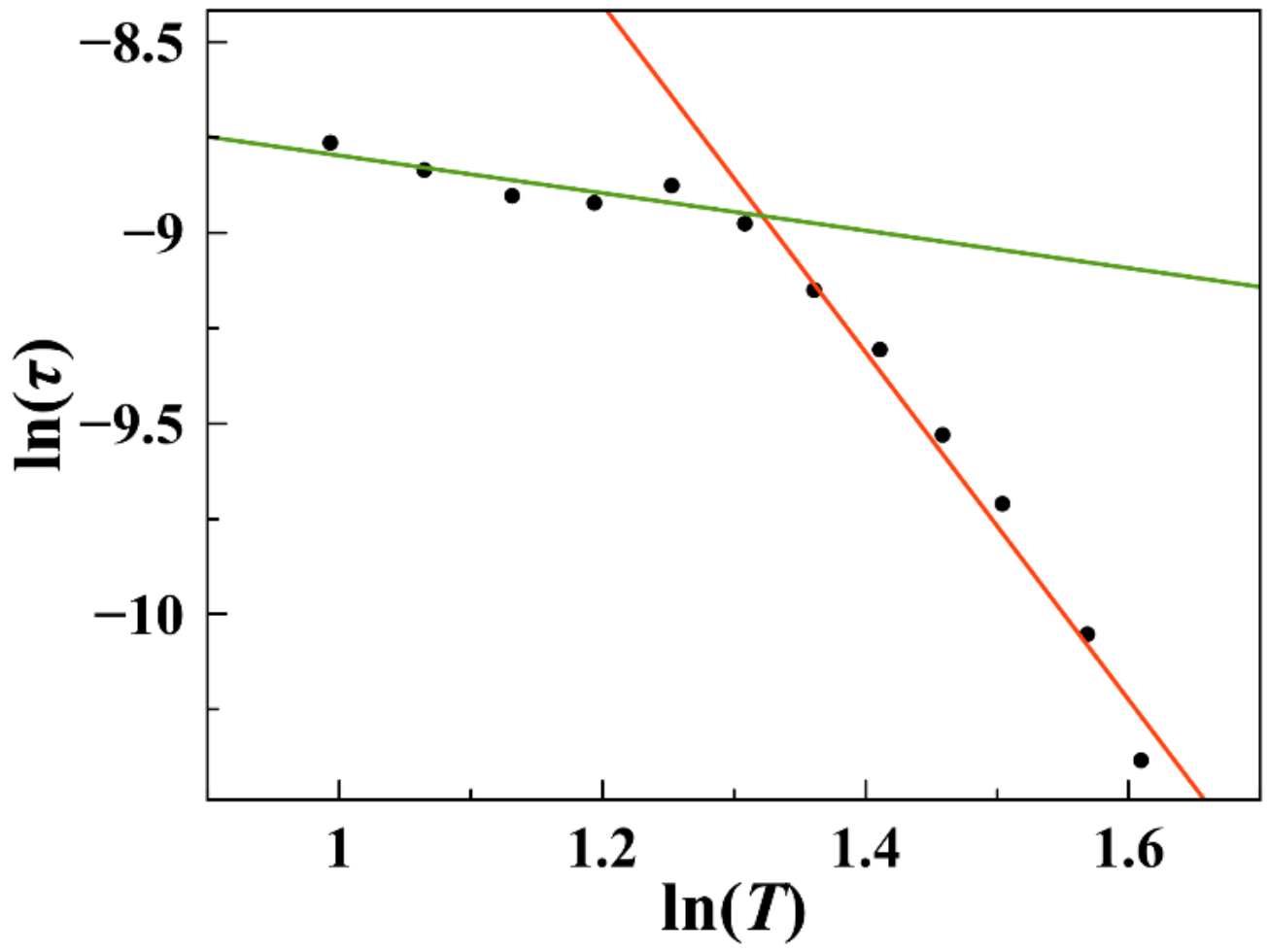

Figure S9: Log-log plot for compound **1**.

Table S3: Best obtained fittings with their corresponding standard deviations of the *ac* data to Eq. 1

	C	*n*	*QT* / s^{-1}
1	0.015(2)	8.8(4)	6565(182)

4- Electron Paramagnetic Resonance

Table S4. The results of CASSCF/NEVPT2 calculations for the molecular fragments of Yb.

	g-tensor of 1st KD	tunnelling rate of 1st KD	ΔE_{1-2} (cm^{-1})
A	0.452703<br>1.078433<br>7.026217	0.255115256274E+00	122.7
B	0.369406<br>0.967773<br>7.142313	0.222798277281E+00	149.1
A(AA)	0.253805<br>0.513071<br>7.346494	0.127777459167E+00	122.6
B(BB)	0.308633<br>0.485901<br>7.604492	0.132378210156E+00	178.16
A(AB)	0.026159<br>0.097330<br>7.547669	0.205755743307E-01	150.75
B(AB)	0.382765<br>0.482066<br>7.676258	0.144089221152E+00	208.8

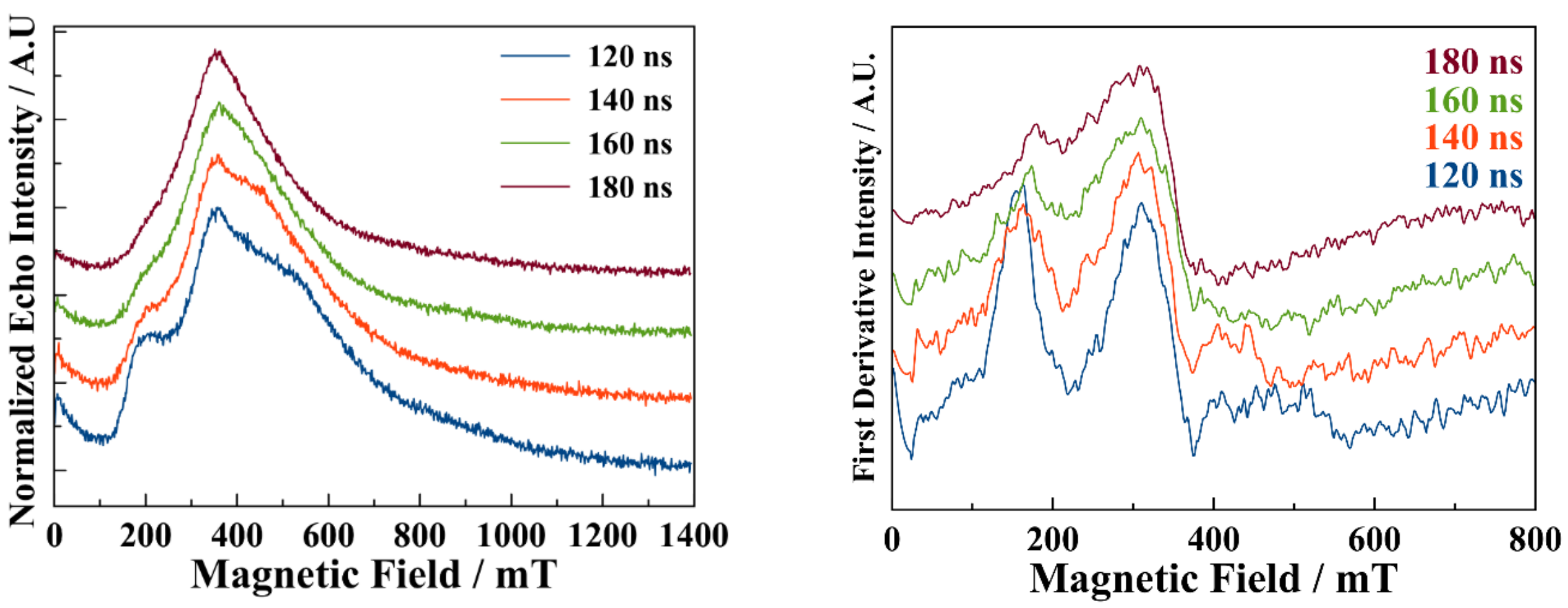

Figure S10: Left: EDFS spectra of $\mathbf{1}_{1\%}$ recorded at a fixed temperature of 7 K and with different interpulse delay time variations. Right: First derivative of the recorded EDFS spectra.

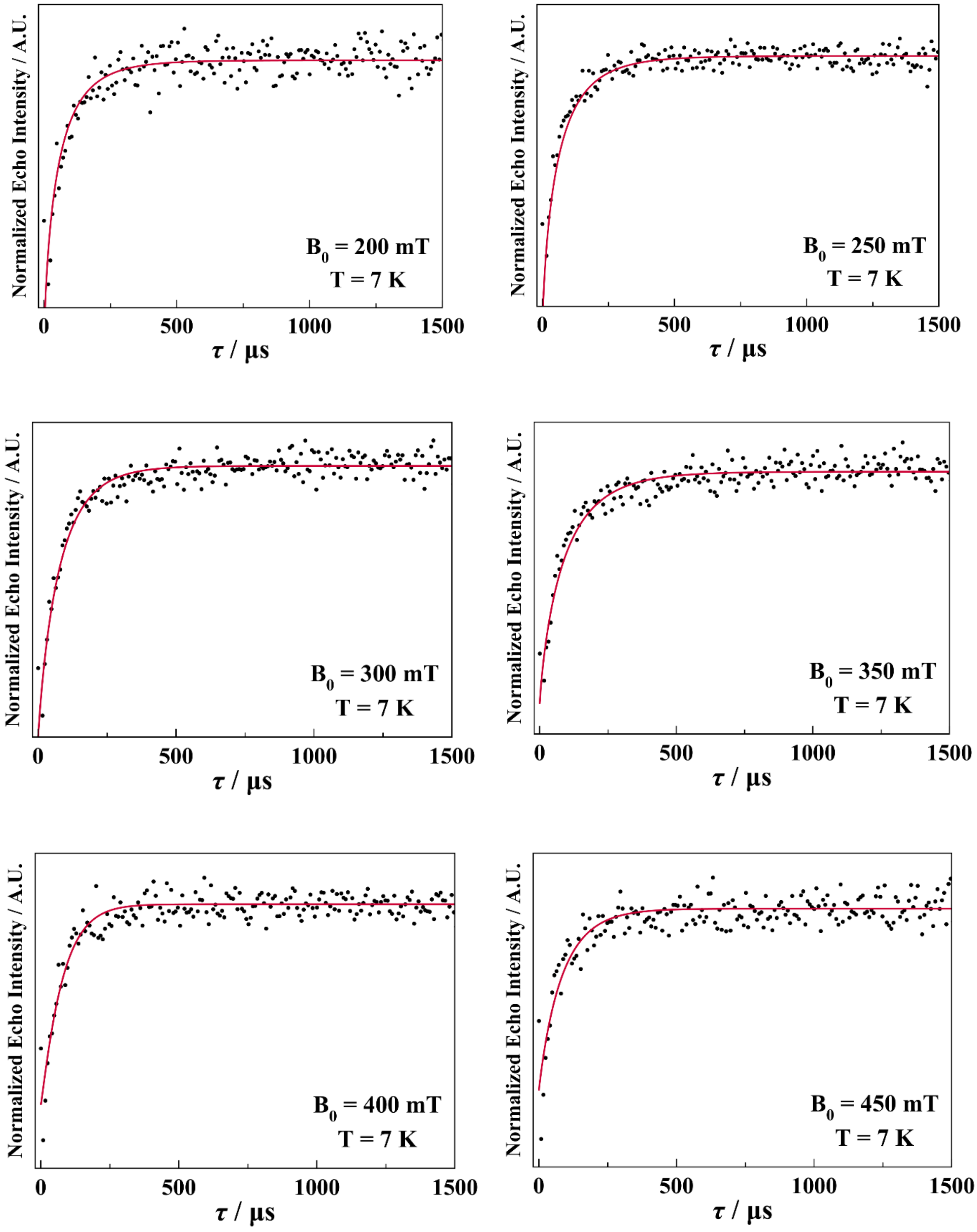
Normalized Echo Intensity / A.U.
B0 = 200 mT
T = 7 K
0
500
1000
1500
τ / μs
Normalized Echo Intensity / A.U.
B0 = 250 mT
T = 7 K
0
500
1000
1500
τ / μs
Normalized Echo Intensity / A.U.
B0 = 300 mT
T = 7 K
0
500
1000
1500
τ / μs
Normalized Echo Intensity / A.U.
B0 = 350 mT
T = 7 K
0
500
1000
1500
τ / μs
Normalized Echo Intensity / A.U.
B0 = 400 mT
T = 7 K
0
500
1000
1500
τ / μs
Normalized Echo Intensity / A.U.
B0 = 450 mT
T = 7 K
0
500
1000
1500
τ / μs

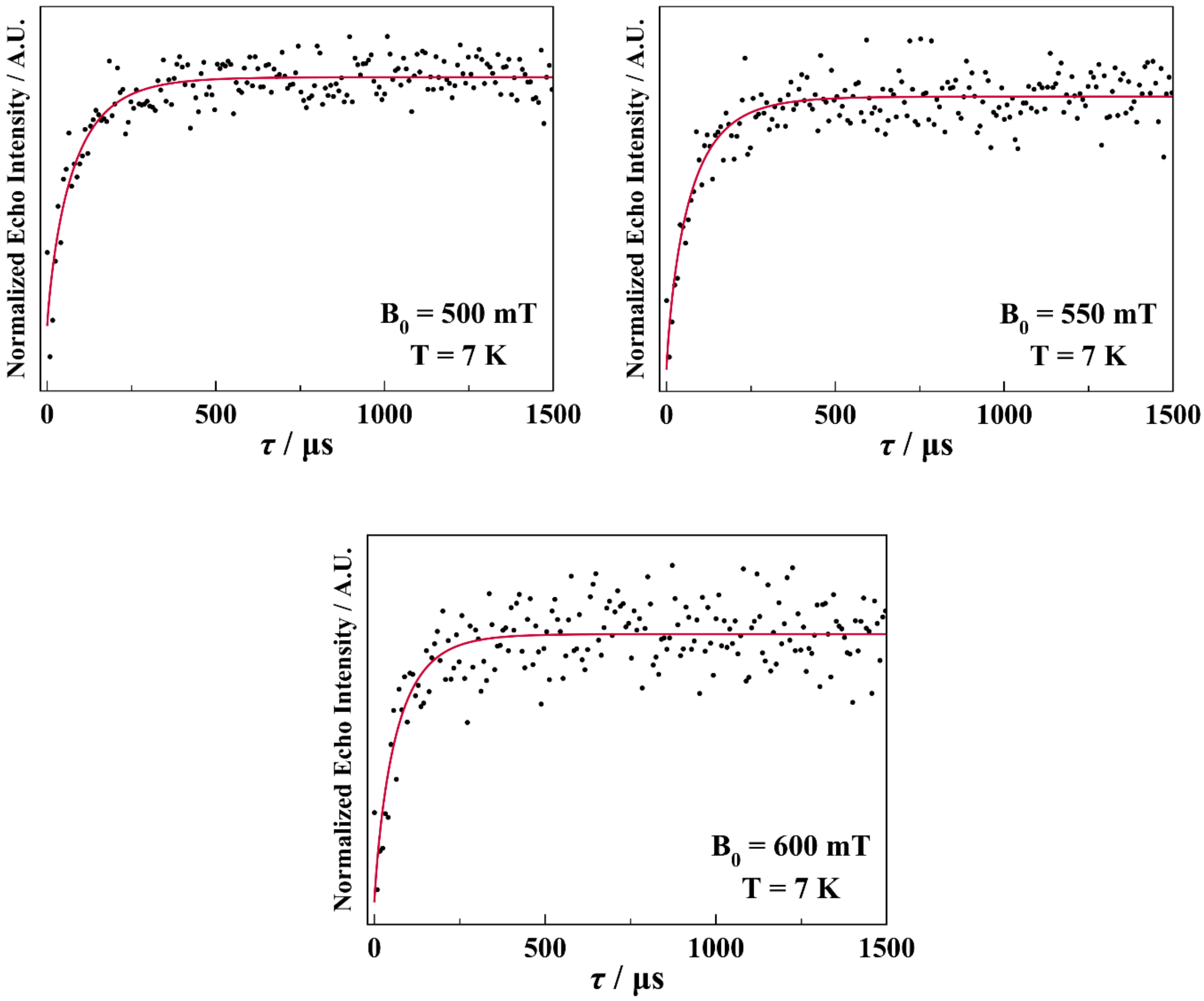

Figure S11: Inversion recovery experiments recorded at different fields and at a fixed temperature of 7 K. Solid lines correspond to the best fits to a stretched exponential.

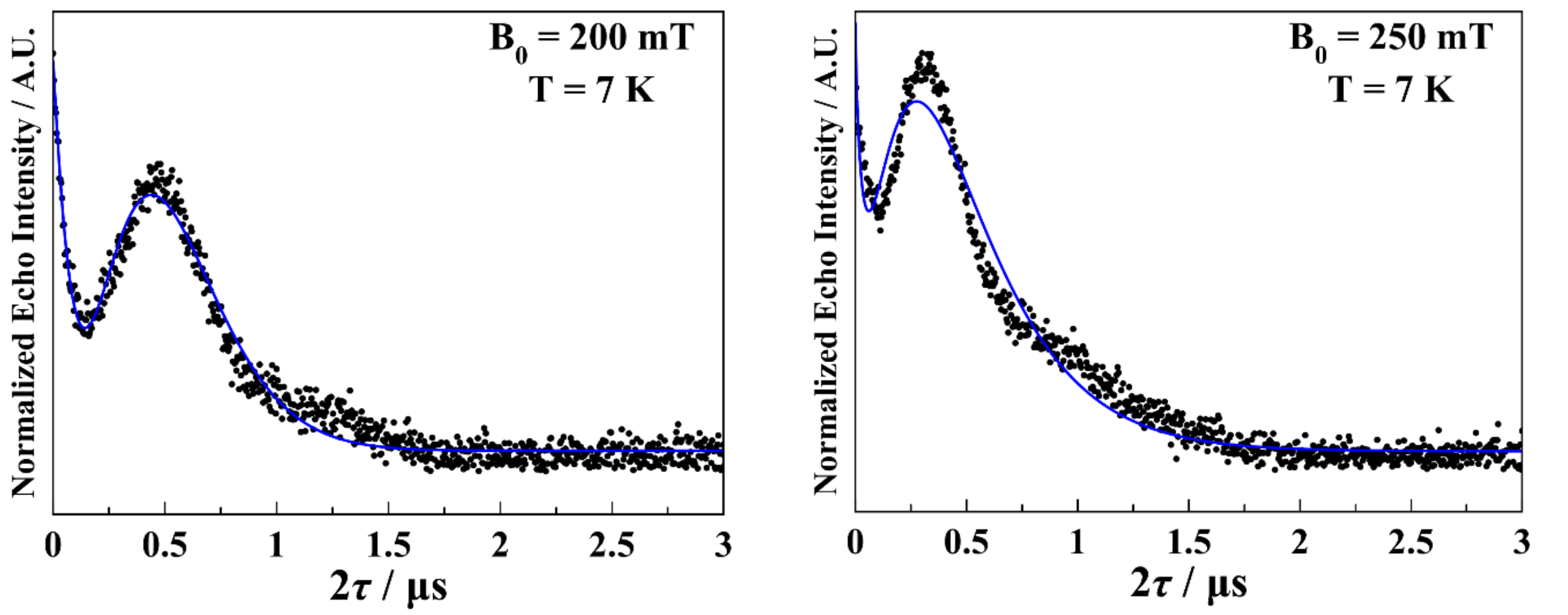

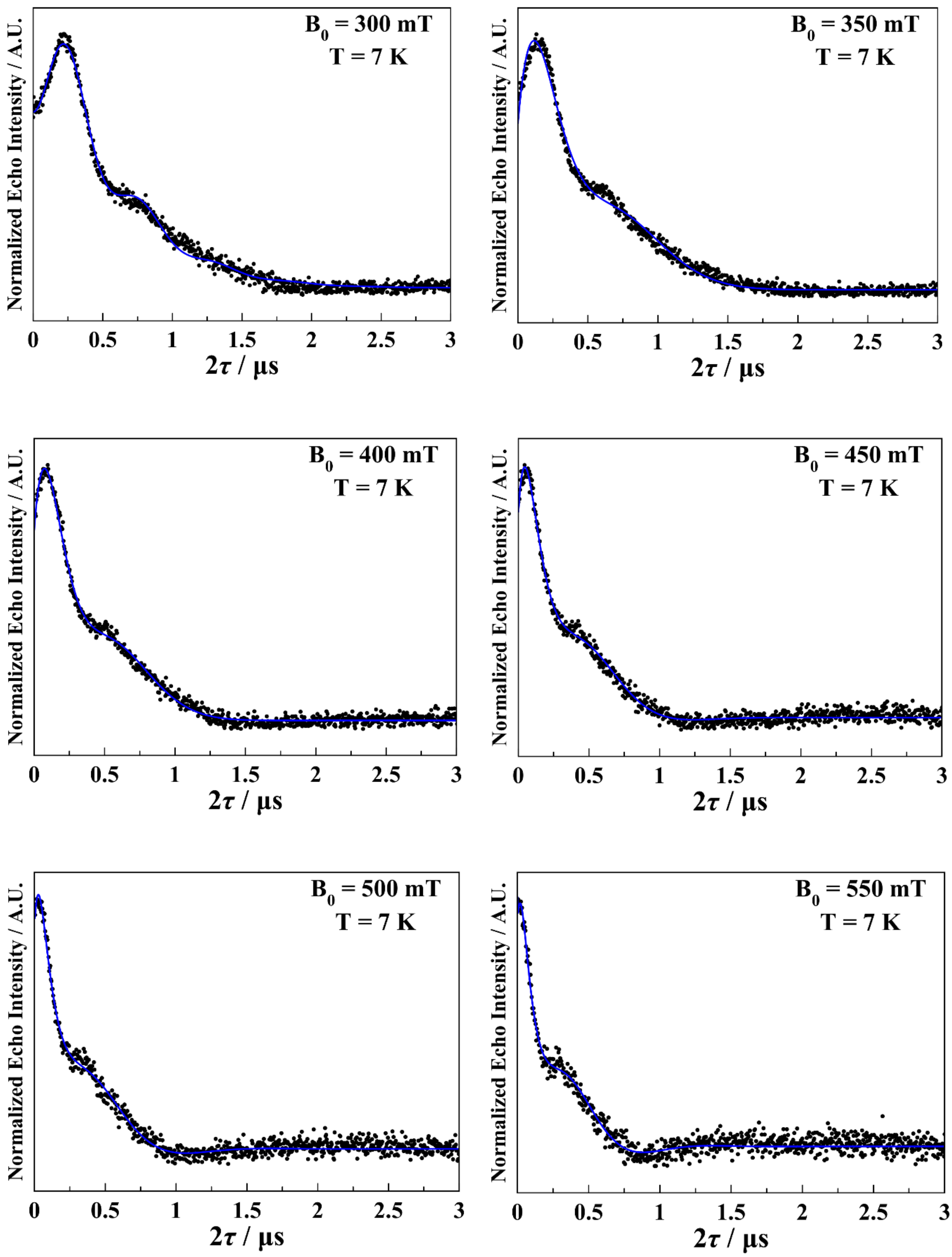
B0 = 300 mT
T = 7 K
B0 = 350 mT
T = 7 K
B0 = 400 mT
T = 7 K
B0 = 450 mT
T = 7 K
B0 = 500 mT
T = 7 K
B0 = 550 mT
T = 7 K
Normalized Echo Intensity / A.U.
2τ / µs
0
0.5
1
1.5
2
2.5
3

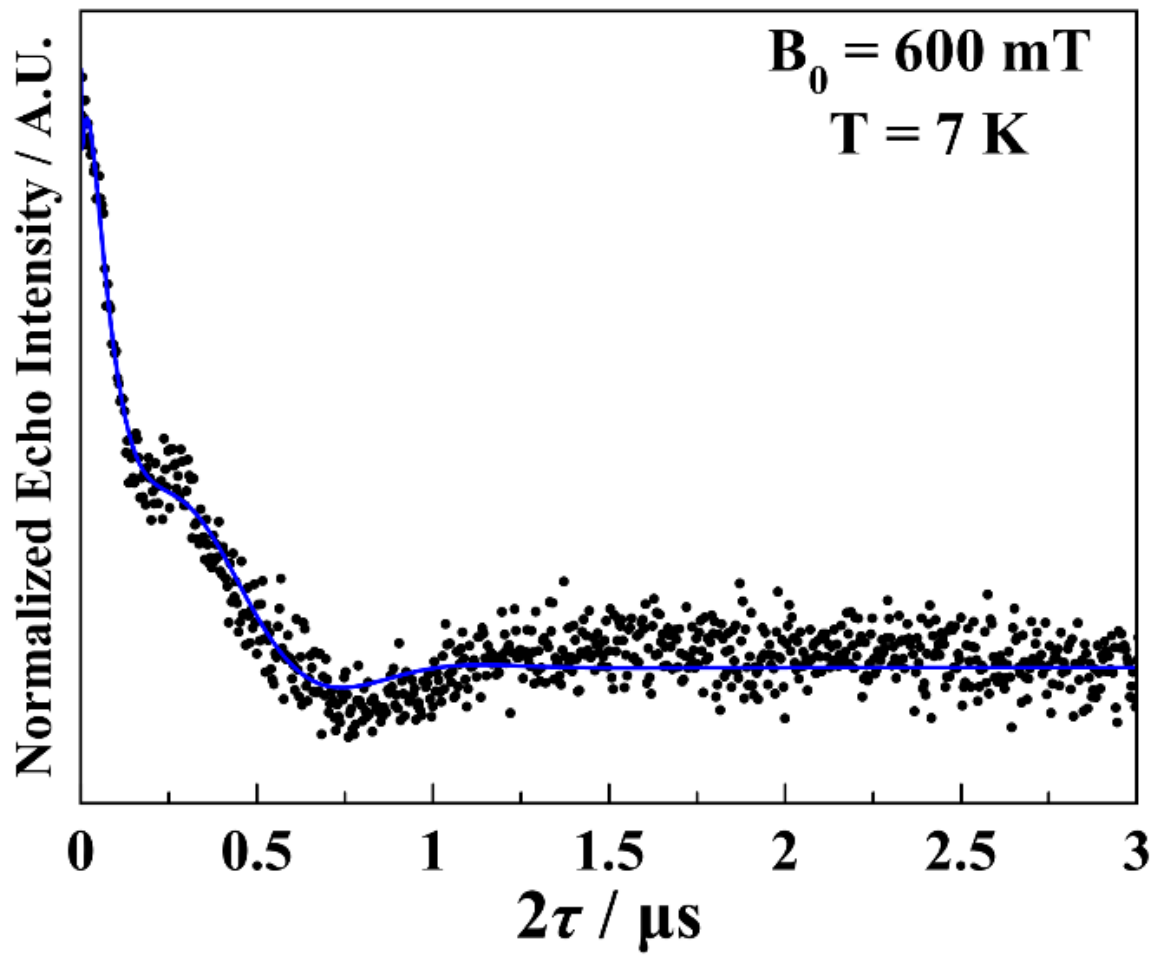

Figure S12: Hahn echo decays recorded at variable magnetic fields at a fixed temperature of 7 K. Solid lines correspond to the best fits to a cosine-modulated stretched exponential decay function.

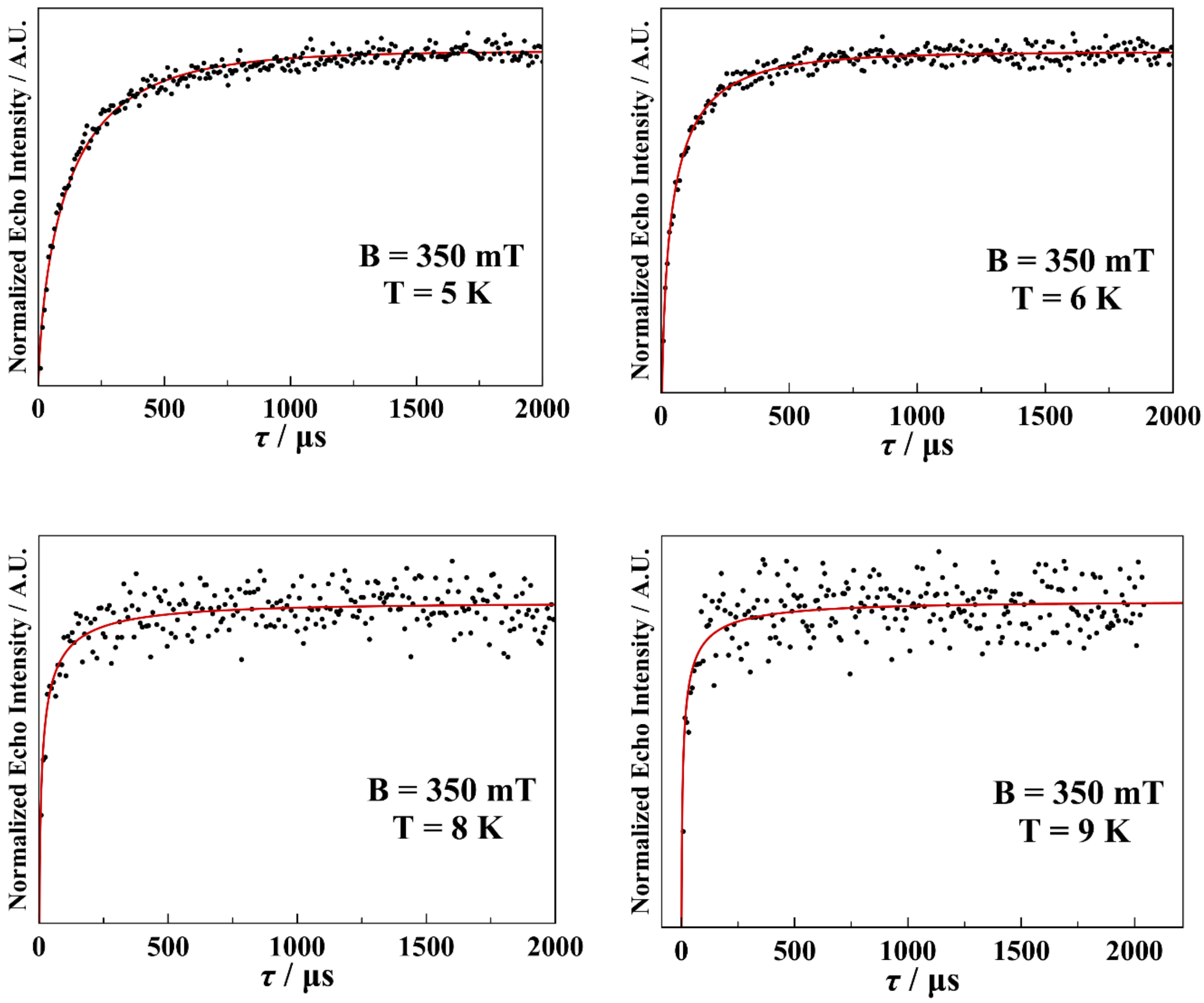

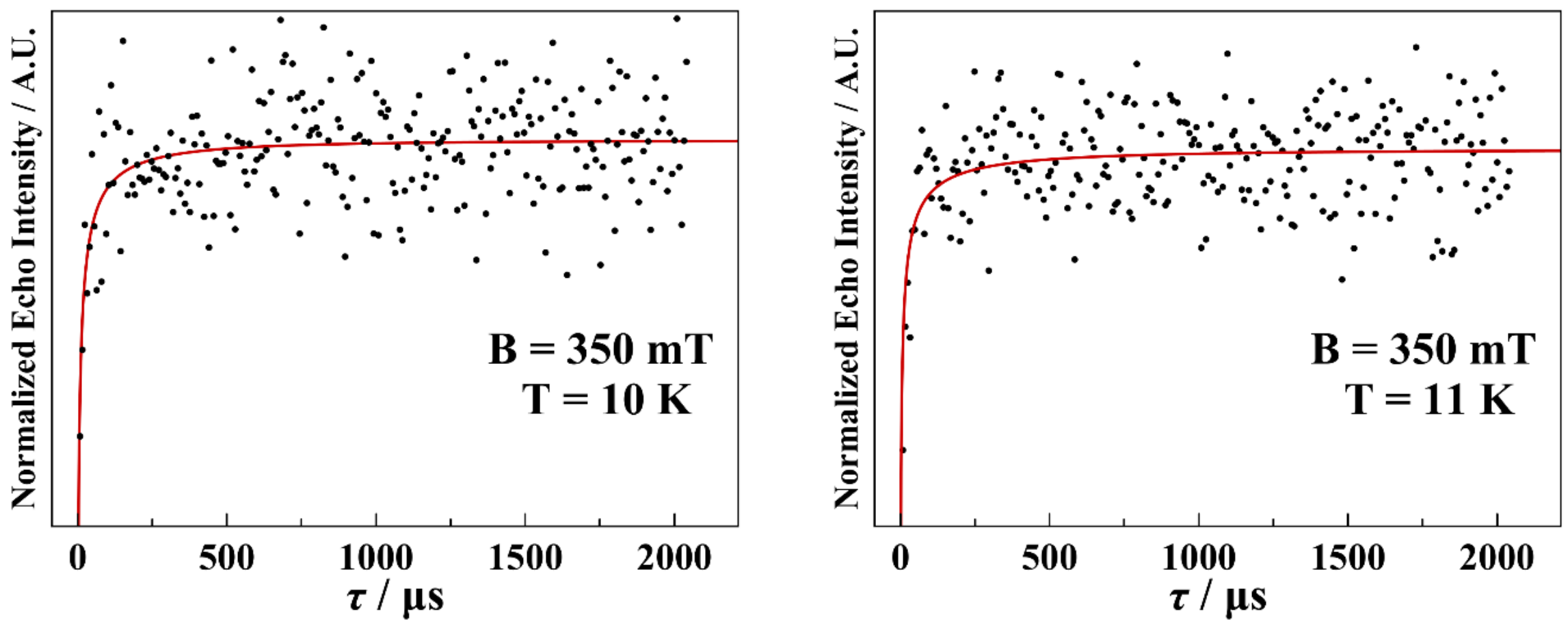

Figure S13: Inversion recovery curves recorded at different temperatures under a static magnetic field of 350 mT. Solid lines correspond to the best fits to a stretched exponential function.

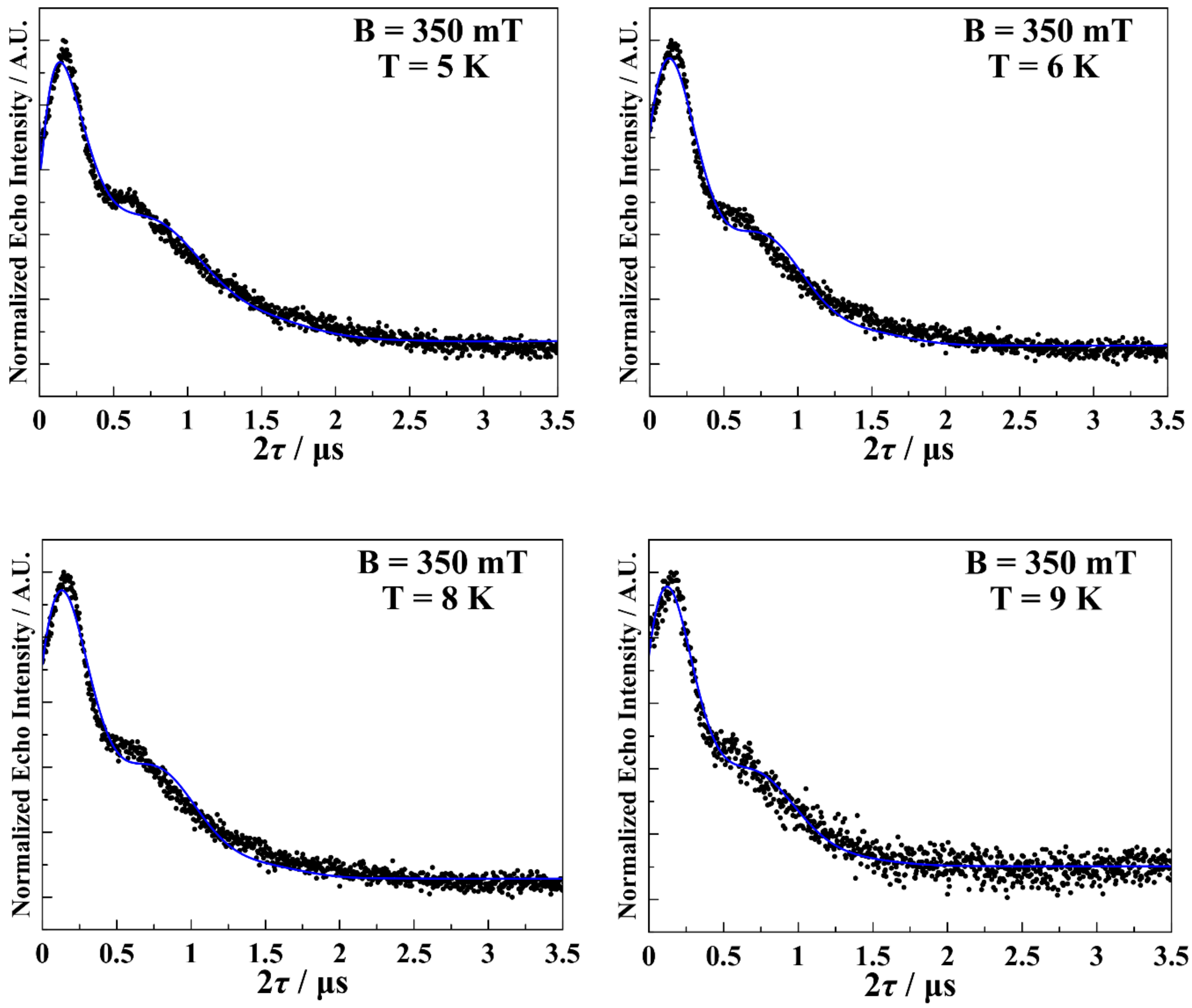

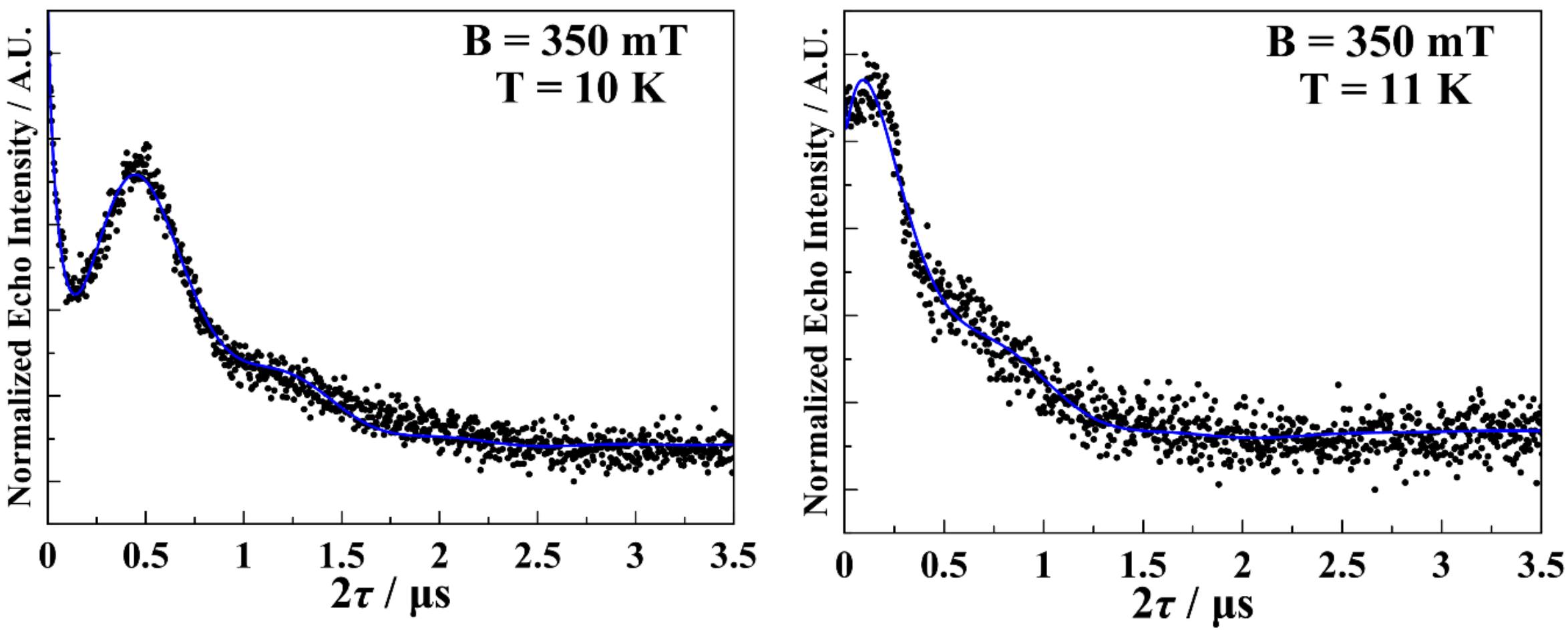

Figure S14: Hahn echo decay curves recorded at different temperatures under a static field of 350 mT. The solid lines correspond to the best fits to a cosine modulated stretched exponential function.

Table S5: Values obtained from fitting inversion recovery curves recorded at different fields at a fixed temperature of 7 K to Eq. 3.

**B / mT**	200	250	300	350	400	450	500	550	600
**A**	0.87(2)	0.92(1)	0.78(3)	0.80(3)	0.76(3)	0.69(4)	0.77(2)	0.85(3)	0.83(4)
**T_1 / μs**	60(2)	61(2)	79(4)	85(3)	79(3)	78(6)	77(4)	69(1)	65(3)
**β_1**	0.72(4)	0.73(3)	0.89(5)	0.79(5)	1.08(9)	0.94(1)	0.84(6)	0.81(6)	0.86(1)
**d**	0.871(3)	0.92(3)	0.92(1)	0.90(3)	0.89(1)	0.881(3)	0.872(4)	0.912(2)	0.788(6)

Table S6: Values obtained from fitting inversion recovery curves recorded at different temperatures under a static field of 350 mT to Eq. 3.

**T / K**	5	6	8	9	10	11
**A**	0.84(1)	1.22(1)	1.77(6)	2.0(1)	2.4(1)	2.7(2)
**T_1 / μs**	122(1)	27.6(3)	2.3(1)	0.88(4)	0.35(4)	0.17(2)
**β_1**	0.64(1)	0.426(6)	0.266(1)	0.23(1)	0.23(1)	0.20(1)
**d**	0.957(2)	0.959(2)	0.903(4)	0.821(6)	0.860(4)	0.782(6)

Table S7: Values obtained from fitting the Hahn echo decays recorded at different fields and at a fixed temperature of 7 K to Eq. 4.

**B / mT**	200	250	300	350	400	450	500	550	600
**A**	13.35(5)	52(2)	11.3(5)	2.3(4)	2.91(4)	7.5(2)	34(2)	8(1)	1.8(1)
**T_m / µs**	0.22(1)	0.38(1)	0.43(2)	0.38(5)	0.26(1)	0.12(4)	0.04(1)	0.03(1)	0.017(3)
**β_m**	0.96(1)	1.426(1)	1.46(1)	1.19(3)	1.13(4)	0.92(3)	0.71(1)	0.61(1)	0.52(4)
**d**	0.044(1)	0.040(1)	0.032(1)	0.026(2)	0.033(2)	0.045(2)	0.057(3)	0.072(2)	0.105(1)
**m_1**	0.803(1)	1.006(4)	0.944(4)	0.820(2)	0.91(3)	1.100(4)	1.17(1)	2.0(2)	13(2)
**m_2**	0.17(1)	0.047(2)	0.065(2)	0.210(3)	0.24(5)	-0.25(3)	-0.24(1)	-0.7(1)	-5.5(6)
**φ_1**	-3.84(2)	-3.475(2)	-3.38(1)	-2.8(2)	-2.9(2)	-3.0(2)	-3.25(8)	-2.5(1)	-2.0(4)
**φ_2**	122.16(4)	123.34(2)	129.93(2)	130.76(6)	143.6(1)	121.8(2)	140.7(1)	141(3)	141(4)
**f_1 / MHz**	0.234(2)	0.17(2)	0.21(2)	0.34(2)	0.45(1)	0.59(2)	0.73(2)	0.90(3)	1.03(5)
**f_2 / MHz**	0.82(2)	0.96(3)	1.16(1)	1.26(4)	1.417(5)	1.55(1)	1.7(1)	1.8(1)	2.0(2)

Table S8: Values obtained along with standard deviations from fitting the Hahn echo decays recorded at different temperatures and at a fixed static field of 350 mT to Eq. 4. The asterisks denote fixed parameters in the fitting.

**T / K**	5	6	7	8	9	10	11
**A**	2.4(5)	2.5(4)	2.3(4)	2.4(6)	3.8(3)	3.9(3)	0.9(2)
**T_m / µs**	0.346(4)	0.336(5)	0.38(5)	0.340(5)	0.30(1)	0.13(3)	0.080(2)
**β_m**	0.80(3)	0.92(4)	1.19(3)	1.00(5)	1.08(3)	0.61(1)	0.54(3)
**d**	0.075(1)	0.056(2)	0.026(2)	0.056(1)	0.10(3)	0.082(1)	0.13(3)
**m_1**	0.90(2)	-0.87(2)	0.820(2)	0.82(3)	-0.86(1)	0.76(1)	-5.0(8)
**m_2**	-0.17(3)	-0.19(4)	0.210(3)	-0.21(4)	-0.16(2)	-0.30(2)	-1.0(3)
**φ_1**	179(3)	-6.0(2)	-2.8(2)	-2.8(2)	-6.2(1)	28.91(6)	-4.79(5)
**φ_2**	127(4)	133(2)	130(6)	133(3)	133(2)	243(7)	133(4)
**f_1 / MHz**	0.34*	0.34*	0.34(2)	0.34*	0.34*	0.34*	0.34*
**f_2 / MHz**	1.26*	1.26*	1.26(4)	1.26*	1.26*	1.26*	1.26*

5- Theoretical calculations

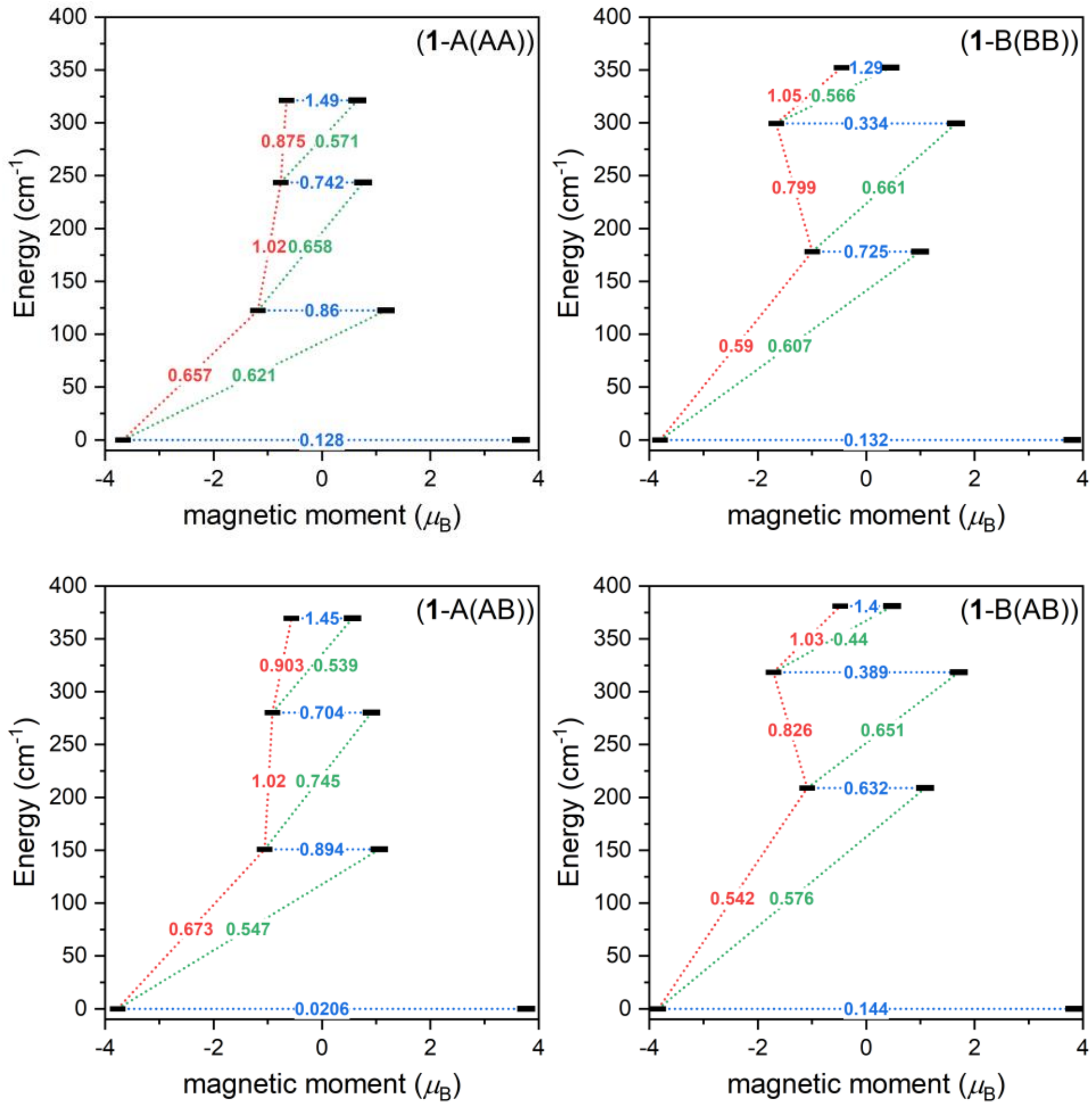

Figure S15: The output of the CASSCF/NEVPT2 calculations for the dinuclear molecular fragments of Yb(III) compound **1 - t**he magnetization reversal barriers.

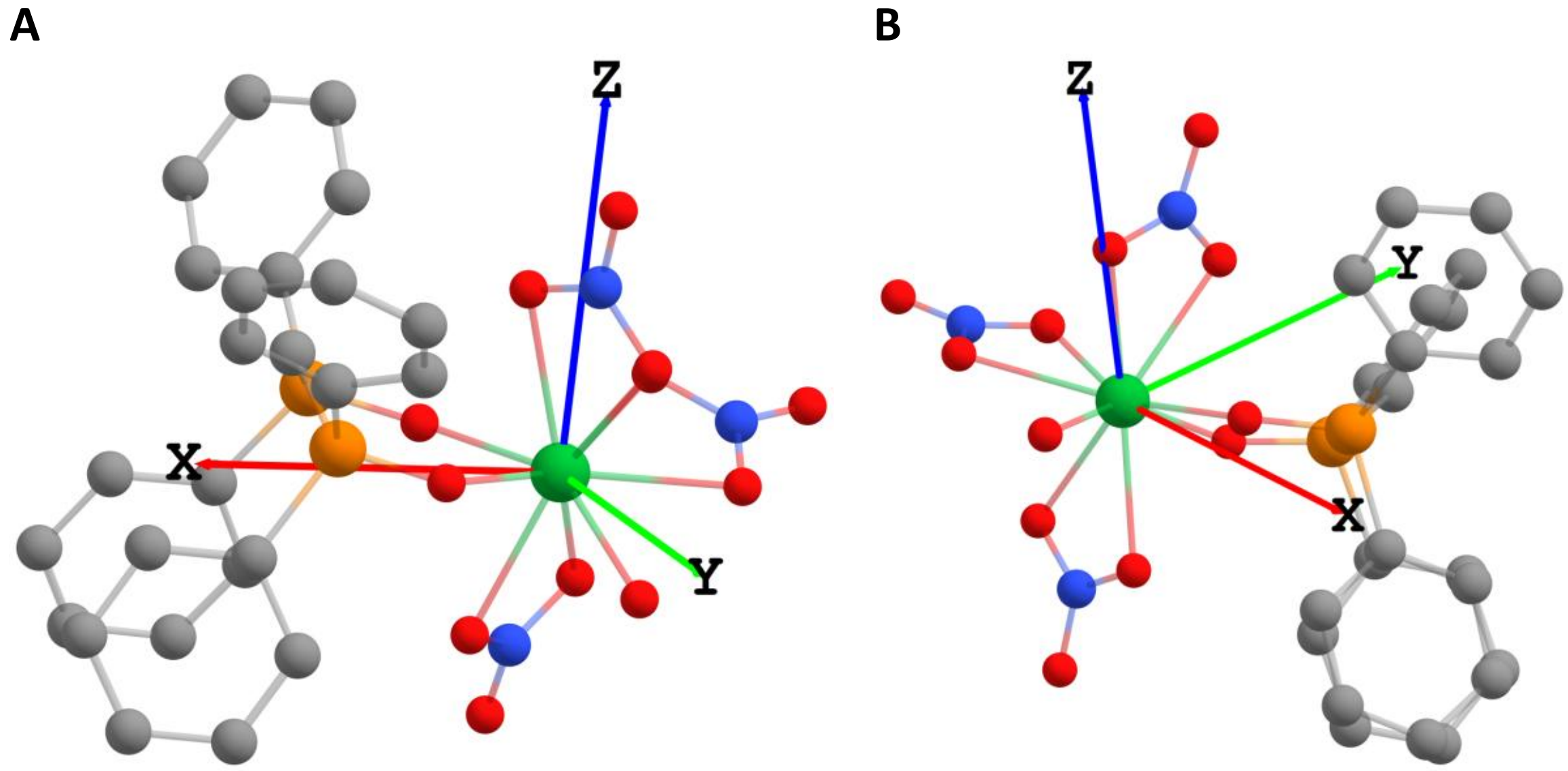

Figure S16: The molecular structures of A (left) and B (right) fragments of **1** overlaid with the principal axes of ground state Kramers' doublet g-tensor (x/y/z-axes colored as red/green/blue arrows) resulting from CASSCF/NEVPT2 calculations.

Table S9: The results of QT-AIM analysis for molecular fragment A of **1** [a]

Bond	Distance (Å)	ρ(**r**)	∇²ρ(**r**)	h_e(**r**)	|V(**r**)|/G(**r**)
Yb1 - O7	2.478	0.0383	0.1633	-0.00028	1.007
Yb1 - O9	2.392	0.0481	0.1983	-0.00292	1.056
Yb1 - O11	2.444	0.0427	0.1773	-0.00167	1.036
Yb1 - O12	2.381	0.0497	0.1998	-0.00350	1.065
Yb1 - O14	2.463	0.0401	0.1694	-0.00074	1.017
Yb1 - O15	2.405	0.0467	0.1924	-0.00262	1.052
Yb1 - O17^{H2O}	2.295	0.0527	0.2631	-0.00023	1.003
Yb1 - O5^{dppmO2}	2.230	0.0625	0.3095	-0.00329	1.041
Yb1 - O6^{dppmO2}	2.252	0.0599	0.2876	-0.00318	1.042

[a] the Laplacian of the electron density (∇²ρ(**r**)), electron kinetic energy (G(**r**)), potential energy density (V(**r**)) and electron energy density (h_e(**r**)).

Table S10: The results of QT-AIM analysis for molecular fragment B of **1** [a]

Bond	Distance (Å)	$\rho(\mathbf{r})$	$\nabla^2\rho(\mathbf{r})$	$h_e(\mathbf{r})$	$\lvert V(\mathbf{r})\rvert/G(\mathbf{r})$
Yb1 - $O8^{NO3}$	2.491	0.0372	0.1580	-0.00010	1.002
Yb1 - $O10^{NO3}$	2.383	0.0488	0.2032	-0.00300	1.056
Yb1 - $O12^{NO3}$	2.379	0.0501	0.2000	-0.00371	1.069
Yb1 - $O13^{NO3}$	2.438	0.0432	0.1781	-0.00168	1.036
Yb1 - $O15^{NO3}$	2.448	0.0419	0.1764	-0.00125	1.027
Yb1 - $O16^{NO3}$	2.422	0.0447	0.1846	-0.00207	1.043
Yb1 - $O18^{H2O}$	2.304	0.0520	0.2594	-0.00020	1.003
Yb1 - $O6^{dppmO2}$	2.258	0.0584	0.2848	-0.00243	1.033
Yb1 - $O7^{dppmO2}$	2.225	0.0635	0.3131	-0.00375	1.046

[a] the Laplacian of the electron density ($\nabla^2\rho(\mathbf{r})$), electron kinetic energy ($G(\mathbf{r})$), potential energy density ($V(\mathbf{r})$) and electron energy density ($h_e(\mathbf{r})$).

Parameters employed to simulate field-swept EPR spectra.

Sys0.S = 1/2;

Sys0.g = [1.93 5.48]; % axial two-g model, third g out of experimental range;

Sys.Nucs = '171Yb,173Yb';

Sys.A = [299.99 299.58; 261.51 313.28];

Table S11. Results of CASSCF/NEVPT2 calculations for molecular geometries of **1**: Energies of Kramers' doublets (KD) originating from $^2F_{7/2}$ ground multiplet of Yb(III) and respective g-factors, decomposition of the wavefunction corresponding to the lowest multiplet with J = 7/2, and angles of g_z-axis of excited Kramers doublets related to the orientation of the ground state g_z-vector (Θ).

a) Calculation for A

KD	E (cm^{-1})	g_x<br>g_y<br>g_z	$\lvert\pm7/2\rangle$	$\lvert\pm5/2\rangle$	$\lvert\pm3/2\rangle$	$\lvert\pm1/2\rangle$	Θ(°)
1	0	0.45257164<br>1.07811990<br>7.02416588	84.3	1.3	14.2	0.3	0.0
2	122.710	0.20443450<br>2.45502458<br>4.88276896	8.7	59.8	26.8	4.7	63.3
3	245.075	0.82256818<br>1.85105496<br>6.38181862	0.8	22.9	17.3	59.1	82.1
4	349.278	0.86153005<br>1.56881122<br>7.02351793	6.2	16	41.9	35.9	91.1

b) Calculation for B

KD	E (cm^{-1})	g_x<br>g_y<br>g_z	$\lvert\pm7/2\rangle$	$\lvert\pm5/2\rangle$	$\lvert\pm3/2\rangle$	$\lvert\pm1/2\rangle$	Θ(°)
1	0	0.36929633<br>0.96749334<br>7.14022788	88.1	0.8	8.2	3.0	0.0
2	149.101	0.15554904<br>1.93628870<br>5.29685427	8.4	33.1	42.6	15.9	66.9
3	308.895	0.70045016<br>1.14071368<br>6.55216994	0.7	31.7	28.4	39.2	76.3
4	383.044	1.37274727<br>2.11823508<br>6.35486656	2.8	34.4	20.8	41.9	96.1

c) Calculation for A(AA)

KD	E (cm^{-1})	g_x g_y g_z	$\|\pm 7/2\rangle$	$\|\pm 5/2\rangle$	$\|\pm 3/2\rangle$	$\|\pm 1/2\rangle$	Θ(°)
1	0	0.25373786<br>0.51292689<br>7.34435237	90.1	0.3	8.1	1.6	0.0
2	122.619	4.69098910<br>3.09602465<br>0.25517482	4.7	47.7	45.3	2.2	48.7
3	243.655	0.41926434<br>1.36194974<br>5.82678794	1.8	28.9	16.2	53.0	78.3
4	321.365	0.95104987<br>1.79137370<br>6.50810368	3.3	23.1	30.3	43.3	90.4

d) Calculation for B(BB)

KD	E (cm^{-1})	g_x g_y g_z	$\|\pm 7/2\rangle$	$\|\pm 5/2\rangle$	$\|\pm 3/2\rangle$	$\|\pm 1/2\rangle$	Θ(°)
1	0	0.30852625<br>0.48574301<br>7.60227663	95.8	0.6	1.4	2.1	0.0
2	178.161	0.16616966<br>2.29626819<br>5.45379447	1.6	28.9	58.6	10.9	73.6
3	299.485	0.52198769<br>1.06707863<br>5.71955171	1.5	55.4	25.4	17.6	54.8
4	352.215	0.71584205<br>2.67199803<br>5.53011411	1.1	15.1	14.5	69.4	92.9

e) Calculation for A(AB)

KD	E (cm^{-1})	g_x<br>g_y<br>g_z	$\|\pm 7/2\rangle$	$\|\pm 5/2\rangle$	$\|\pm 3/2\rangle$	$\|\pm 1/2\rangle$	Θ(°)
1	0	0.02614016<br>0.09731328<br>7.54546915	93.8	0.1	4.3	1.8	0.0
2	150.756	4.59077512<br>3.38975906<br>0.41835300	2.1	43.1	52.8	2.0	37.8
3	280.310	0.51586622<br>1.20837398<br>5.74490709	2.0	35.9	19.2	43.0	75.5
4	369.530	0.85693770<br>1.58863487<br>6.69734888	2.0	20.9	23.8	53.2	92.7

f) Calculation for B(AB)

KD	E (cm^{-1})	g_x<br>g_y<br>g_z	$\|\pm 7/2\rangle$	$\|\pm 5/2\rangle$	$\|\pm 3/2\rangle$	$\|\pm 1/2\rangle$	Θ(°)
1	0	0.3826310<br>0.4819042<br>7.6740211	96.9	0.6	0.9	1.6	0.0
2	208.807	0.06574038<br>2.16036316<br>5.49391661	0.9	28.5	63.0	7.5	68.8
3	318.458	0.49088694<br>1.21713796<br>5.70197911	1.2	56.3	20.5	22.0	54.3
4	380.854	0.89787783<br>2.46879886<br>5.93894874	1.1	14.5	15.6	68.8	93.2
